# Multiple linear regression to estimate time-frequency electrophysiological responses in single trials

**DOI:** 10.1016/j.neuroimage.2015.01.062

**Published:** 2015-05-01

**Authors:** L. Hu, Z.G. Zhang, A. Mouraux, G.D. Iannetti

**Affiliations:** a Key Laboratory of Cognition and Personality (Ministry of Education) and Faculty of Psychology, Southwest University, Chongqing, China; b Department of Electrical and Electronic Engineering, The University of Hong Kong, Hong Kong, China; c Institute of Neurosciences (IoNS), Université catholique de Louvain, Brussels, Belgium; d Department of Neuroscience, Physiology and Pharmacology, University College London, UK; e School of Chemical and Biomedical Engineering and School of Electrical and Electronic Engineering, Nanyang Technological University, Singapore

**Keywords:** Time-frequency analysis, Single-trial analysis, Event-related desynchronization (ERD), Event-related synchronization (ERS), Multiple linear regression, Neuronal oscillations

## Abstract

Transient sensory, motor or cognitive event elicit not only phase-locked event-related potentials (ERPs) in the ongoing electroencephalogram (EEG), but also induce non-phase-locked modulations of ongoing EEG oscillations. These modulations can be detected when single-trial waveforms are analysed in the time-frequency domain, and consist in stimulus-induced decreases (event-related desynchronization, ERD) or increases (event-related synchronization, ERS) of synchrony in the activity of the underlying neuronal populations. ERD and ERS reflect changes in the parameters that control oscillations in neuronal networks and, depending on the frequency at which they occur, represent neuronal mechanisms involved in cortical activation, inhibition and binding. ERD and ERS are commonly estimated by averaging the time-frequency decomposition of single trials. However, their trial-to-trial variability that can reflect physiologically-important information is lost by across-trial averaging. Here, we aim to (1) develop novel approaches to explore single-trial parameters (including latency, frequency and magnitude) of ERP/ERD/ERS; (2) disclose the relationship between estimated single-trial parameters and other experimental factors (e.g., perceived intensity). We found that (1) stimulus-elicited ERP/ERD/ERS can be correctly separated using principal component analysis (PCA) decomposition with Varimax rotation on the single-trial time-frequency distributions; (2) time-frequency multiple linear regression with dispersion term (TF-MLR_d_) enhances the signal-to-noise ratio of ERP/ERD/ERS in single trials, and provides an unbiased estimation of their latency, frequency, and magnitude at single-trial level; (3) these estimates can be meaningfully correlated with each other and with other experimental factors at single-trial level (e.g., perceived stimulus intensity and ERP magnitude). The methods described in this article allow exploring fully non-phase-locked stimulus-induced cortical oscillations, obtaining single-trial estimate of response latency, frequency, and magnitude. This permits within-subject statistical comparisons, correlation with pre-stimulus features, and integration of simultaneously-recorded EEG and fMRI.

## Highlights

• ERP/ERD/ERS are reliably isolated using PCA + Varimax rotation on single-trial TFDs.
• TF-MLR_d_ enhances the SNR of ERP/ERD/ERS in single trials.
• TF-MLR_d_ provides an unbiased estimation of single-trial parameters of ERP/ERD/ERS.
• Availability of single-trial estimates permits within-subject statistical comparison.

## Introduction

The human electroencephalogram (EEG) and magnetoencephalogram (MEG) largely reflect synchronous changes of slow postsynaptic potentials occurring within a large number of similarly oriented cortical pyramidal neurons (Nunez and Srinivasan, 2006). Brisk sensory, motor or cognitive events can elicit transient changes in the ongoing EEG activity. Such changes are commonly detected as event-related potentials (ERPs) that are both time-locked and phase-locked to the stimulus onset (Mouraux and Iannetti, 2008; Pfurtscheller and Lopes da Silva, 1999). The same events can also induce non-phase-locked modulations of ongoing oscillatory EEG activity, consisting in transient decreases (event-related desynchronization, ERD) or increases (event-related synchronization, ERS) of EEG power, usually confined to a specific frequency band. The functional significance of ERD and ERS differs according to the frequency band in which they occur. For example, within the alpha band (frequencies ranging from 8 to 12 Hz) ERD and ERS have been suggested to reflect cortical activation and cortical deactivation, respectively (reviewed in Pfurtscheller and Lopes da Silva, 1999). In contrast, ERS in the gamma band (frequencies > 30 Hz) has been suggested to reflect the formation of transient cortical assemblies and thus to play a role in cortical integration (Rodriguez et al., 1999; Tallon-Baudry et al., 1997).

Similarly to ERPs, the magnitude of ERD/ERS is often several factors smaller than the magnitude of the background EEG activity (Hu et al., 2010). To enhance the signal-to-noise ratio (SNR) of ERD/ERS, across-trial averaging of time-frequency decompositions (Pfurtscheller and Lopes da Silva, 1999) is the most widely used approach. This approach is based on the expression of signal power within a specific frequency band of interest, as a function of time, or the joint time-frequency distribution (TFD) of the power changes obtained using different approaches, like the windowed Fourier transform or the continuous wavelet transform (Mouraux and Iannetti, 2008). Unfortunately, all the dynamic information concerning across-trial variability of ERP/ERD/ERS is lost by this across-trial averaging procedure (Mouraux and Iannetti, 2008). This variability may reflect important factors such as changes in stimulus intensity, habituation (Iannetti et al., 2008; Ohara et al., 2004; Stancak et al., 2003), as well as fluctuations in vigilance, expectation, task complexity, and emotional or attentional status (Mu et al., 2008; Ploner et al., 2006). The availability of robust signal processing techniques that can quantify latency, frequency, and magnitude of stimulus-induced oscillations in single-trials would allow exploring such physiologically-relevant information in a range of analyses. These include within-subject statistical comparisons, correlation with pre-stimulus features, integration of simultaneously-recorded EEG and functional magnetic resonance imaging data (fMRI) (Debener et al., 2006).

A variety of advanced methods for single-trial analysis of phase-locked ERPs have been proposed (Barbati et al., 2008; Barbati et al., 2006; Hu et al., 2010; Hu et al., 2011b; Jung et al., 2001; Mayhew et al., 2010; Mayhew et al., 2006; Porcaro et al., 2008; Porcaro et al., 2009; Porcaro et al., 2010; Quiroga, 2000; Quiroga and Garcia, 2003; Tang et al., 2005; Tecchio et al., 2007). An important step to improve the effectiveness of extracting single-trial information is enhancing the low SNR of ERPs. This can be obtained by exploiting the spatial information of multielectrode EEG recordings using spatial-temporal filtering based on blind source separation (BSS) methods (e.g., independent component analysis [ICA] and second-order blind identification) to isolate stimulus-related responses from background EEG (Bingham and Hyvarinen, 2000; Hyvarinen, 1999; Hyvarinen and Oja, 2000; Jung et al., 2001; Makeig et al., 1997; Tang et al., 2005). Time-frequency filtering based, for example, on a continuous or discrete wavelet transform (Hu et al., 2010; Hu et al., 2011b; Jongsma et al., 2006; Mouraux and Plaghki, 2004; Quiroga, 2000; Quiroga and Garcia, 2003) can also be used to isolate effectively stimulus-related, phase-locked responses from background EEG and non-cerebral artefacts. In a previous study aiming at measuring single-trial ERP features, Mayhew et al. (2006) suggested the use of a multiple linear regression method to estimate ERP latencies and amplitudes. This approach was later extended by including a dispersion term to increase the accuracy and the number of estimated features (Hu et al., 2011a). Importantly, most of the available single-trial analysis methods were developed to estimate stimulus-evoked phase-locked responses in the time domain (i.e., ERPs). Therefore, these methods are entirely blind to stimulus-induced non-phase-locked modulations of ongoing EEG oscillations (i.e., ERD and ERS). Extending these approaches to explore the dynamic information of stimulus-induced non-phase-locked activity at a single-trial level constitutes the objective of the present study.

Laser-evoked potentials (LEPs) are considered the best tool to assess the function of nociceptive pathways, and are widely used in both physiological and clinical studies (e.g., Bromm and Treede, 1991; Cruccu et al., 2008; Iannetti et al., 2001). When applied onto the skin, brief laser heat pulses excite selectively Aδ and C fibre free nerve endings in the superficial epidermal layers without coactivating Aβ mechanoreceptors (Bromm and Treede, 1984; Carmon et al., 1976), and EEG responses have been shown to be related to the activation of the spinothalamic tract (Iannetti et al., 2003; Treede et al., 2003). Latency, amplitude and morphology of LEPs exhibit an especially high across-trial variability (Hu et al., 2011a; Iannetti et al., 2005a; Purves and Boyd, 1993), most probably due to a unique combination of peripheral (e.g., time-dependent fluctuations in baseline skin temperature, variability in the number of activated nociceptive fibres, variability in conduction velocity resulting in differences in the spatial summation at central synapses) and cognitive factors (e.g., fluctuations in vigilance, attentional focus and task strategy) (Baumgartner et al., 2005; Lee et al., 2009; Legrain et al., 2003). Therefore, laser-evoked EEG responses, adopted in the current study, represent an interesting model to develop novel approaches to estimate time-frequency features at single-trial level, with potential applications for basic and clinical research.

We describe a novel approach to measure different parameters (latency, frequency, and magnitude) of non-phase-locked time-frequency responses in single trials. Briefly, this approach consists of two steps. First, we used a principal component analysis (PCA) decomposition with Varimax rotation to isolate different response features (i.e., ERP, ERD and ERS) from single-trial TFDs in the time-frequency domain. Second, we used a time-frequency multiple linear regression with a dispersion term (TF-MLR_d_) to estimate latency, frequency and magnitude of ERP, ERD and ERS in each single trial. To validate our approach, we applied it to both real and simulated EEG datasets.

## Methods

### Subjects, experimental paradigm and EEG recording

EEG data were collected from ten healthy volunteers (4 females) aged from 22 to 36 years (29.7 ± 4.6, mean ± SD). All participants gave written informed consent, and the local ethics committee approved the procedures.

Noxious radiant-heat stimuli were generated by an infrared neodymium yttrium aluminium perovskite (Nd:YAP) laser with a wavelength of 1.34 μm (Electronical Engineering, Italy). These laser pulses activate directly nociceptive terminals in superficial skin layers (Baumgartner et al., 2005; Iannetti et al., 2006). Laser pulses were directed to the dorsum of the right hand and a He–Ne laser pointed to the area to be stimulated. The laser pulse was transmitted via an optic fibre and focused by lenses to a spot diameter of approximately 7 mm (38 mm^2^) at the target site. The duration of the laser pulses was 4 ms. Three different energies of stimulation were used (E1: 3.5 ± 0.7 J; E2: 4 ± 0.8 J; E3: 4.5 ± 0.7 J). With these parameters laser pulses elicit a clear pinprick sensation, related to the activation of Aδ skin nociceptors (Iannetti et al., 2006) and result in subjective reports of a range of perceived intensities. After each stimulus, the laser beam target was shifted by approximately 10 mm in a random direction, to avoid nociceptor fatigue and sensitization. The laser beam was controlled by a computer that used two servo-motors (HS-422; Hitec RCD, USA; angular speed, 60°/160 ms) to orient it along two perpendicular axes (Lee et al., 2009).

EEG data were collected in three recording blocks with different stimulus energies, which were counterbalanced across subjects. Participants were seated in a comfortable chair and wore protective goggles. They were asked to focus their attention on the stimuli, relax their muscles and keep their eyes open and gaze slightly downward. Acoustic isolation was ensured using earplugs and headphones. Both the laser beam and the controlling motors were completely screened from the view of the participants. The experiment consisted of three blocks of 60 trials, with an inter-stimulus interval ranging between 20 and 25 s. Between 3 and 6 s after each stimulus, participants were asked to rate verbally the intensity of the sensation evoked by the stimulus, using a numerical scale ranging from 0 to 10, where 0 was “no pain” and 10 was “pain as bad as it could be” (Jensen and Karoly, 2001).

The EEG was recorded using 32 Ag–AgCl electrodes placed on the scalp according to the International 10–20 system, using the nose as reference. Electrode impedances were kept < 5 kΩ. To monitor ocular movements and eye blinks, electro-oculographic (EOG) signals were simultaneously recorded from two surface electrodes, one placed over the lower eyelid, the other placed 1 cm lateral to the outer corner of the orbit. Signals were amplified and digitized using a sampling rate of 1024 Hz and a precision of 12 bits, giving a resolution of 0.195 μV digit^− 1^ (System Plus; Micromed, Italy).

### EEG data preprocessing

EEG data were imported and preprocessed using Letswave (http://nocions.webnode.com/letswave/), and EEGLAB (Delorme and Makeig, 2004), an open source toolbox running under the MATLAB environment. All statistical analyses were carried out using SPSS 16.0 (SPSS Inc., Chicago, IL).

Continuous EEG data were down-sampled to 500 Hz and band-pass filtered between 1 and 30 Hz. EEG epochs were extracted using a window analysis time of 1500 ms (500 ms before and 1000 ms after the laser stimulus) and baseline corrected using the pre-stimulus time interval. To test the possible bias in the automated single-trial detection method, the same number of trials of resting EEG (4000 ms to 2500 ms pre-stimulus) were extracted from the dataset of each subject.

Trials contaminated by eye-blinks and movements were corrected using an ICA algorithm (Delorme and Makeig, 2004; Jung et al., 2001; Makeig et al., 1997). EEG epochs were then visually inspected and trials contaminated by artefacts due to gross movements were removed. In all datasets, individual eye movements, showing a large EOG channel contribution and a frontal scalp distribution, were clearly seen in the removed independent components.

After these pre-processing steps, 58 ± 2 epochs remained for the automated analysis for each subject (577 for all subjects). Similarly, 58 ± 2 epochs of resting EEG were kept for testing detection bias.

### EEG data analysis: time-frequency feature (TF-feature) separation

A time-frequency distribution of each single EEG epoch was calculated using the continuous wavelet transform (CWT) (Mouraux et al., 2003; Mouraux and Iannetti, 2008). The explored frequencies ranged from 1 to 30 Hz in steps of 1 Hz, and the explored latencies between − 500 and 1000 ms in steps of 2 ms. For each estimated frequency, the magnitude of the power spectrum was baseline-corrected by subtracting the average power of the signal in the time-interval between − 400 and − 100 ms, which avoids the positive bias introduced by the percentage approach (Hu et al., 2014). The result of CWT is an expression of the oscillation magnitude (in μV) as a function of time and frequency, including both phase-locked (ERP) and non-phase-locked (ERD/ERS) brain responses (Mayhew et al., 2010; Mouraux and Iannetti, 2008). To distinguish between phase-locked and non-phase-locked EEG responses, we calculated, in each subject, the phase-locking value (PLV; Lachaux et al., 1999), as follows:

(1) $$PLV\left(t,f\right)=\left|\frac{1}{N}\sum_{n=1}^{N}\frac{F_{n}\left(t,f\right)}{\left|F_{n}\left(t,f\right)\right|}\right|$$

where *N* is the number of trials, and *F*(*t*,*f*) is the complex time-frequency estimate at each point (*t*,*f*) of the single-trial EEG time course.

#### PCA separation

**Fig. 1:**
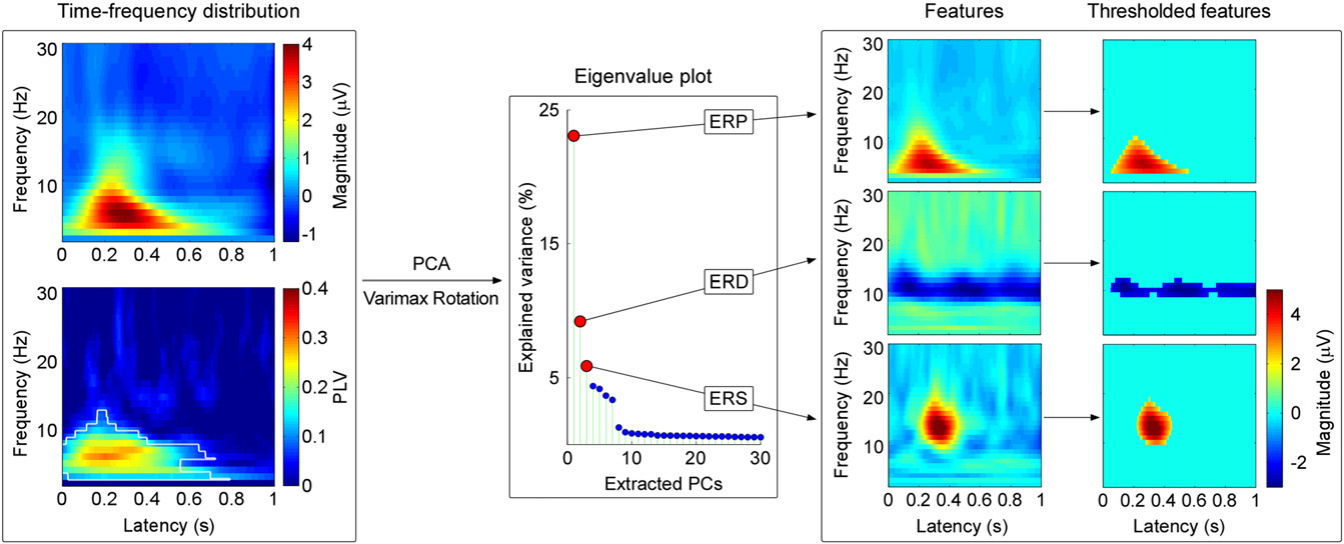
Time-frequency feature isolation using PCA decomposition with Varimax rotation. Left: Group-level TFDs of laser-elicited responses at electrode Cz. x-axis: latency (s); y-axis: frequency (Hz). Both TFDs (top: Magnitude; bottom: PLV) were averaged across all single-trial TFDs of all subjects, after subtracting the baseline. As compared to the baseline, significant differences in PLVs are outlined in white (bootstrapping test with FDR correction), which indicate that only the ERP response was phase-locked to stimulus onset, while the other TFD responses were not. Middle: The eigenvalue plot shows the variance explained by the first 30 extracted PCs. The first three PCs explained the largest amount of variance in the data (23.1%, 9.2% and 5.9%, corresponding to ERP, ERD and ERS responses), while the explained variance for any of the remaining PCs was less than 5%. Right: The first three PCs correspond to the ERP, ERD and ERS in the time-frequency plane, respectively. The thresholded TFDs were obtained by applying a two-SD cut-off. The amount of background EEG noise was remarkably reduced, while the regions corresponding to ERP/ERS/ERD responses were clearly preserved. The ERP region was located at 50–550 ms post-stimulus (in time) and 3–10 Hz (in frequency); the ERD region at 50–1000 ms and 9–12 Hz; the ERS region at 217–447 ms and 10–19 Hz in frequency.

In order to separate physiologically relevant TF-features (i.e., ERP, ERD, and ERS) within the TFDs of single-trial laser-evoked EEG responses measured from Cz-nose, we performed a PCA decomposition with Varimax rotation (Bernat et al., 2005; Dien, 2010; Kayser and Tenke, 2003), as successfully implemented in several recent studies (Bernat et al., 2007; Bernat et al., 2005; Mayhew et al., 2010). This approach allows the separation of physiologically distinct EEG activities that are contained in the time-frequency plane. The procedure of PCA with Varimax rotation consists in the following five steps (summarized in Fig. 1) (Bernat et al., 2005; Mayhew et al., 2010).

(1) *Data concentration*. The TFD of each single trial was re-arranged as a vector, and all vectors from all single trials across all subjects were stacked sequentially to form a single matrix. In this study, we re-arranged the time-frequency matrices from K_T_ × N_T_ × N_F_ (three dimensions: trial numbers of all subjects × time points × frequency points) to K_T_ × N_TF_ (two dimensions: trial numbers × time-frequency points).
(2) *PCA decomposition of the covariance matrix*. The matrix generated in step 1 was decomposed into a set of principal components (PCs) by PCA.
(3) *Varimax rotation*. These PCs were further rotated using the Varimax algorithm, which maximizes the sum of the variances of the squared loadings so that the matrix can be optimally described by a linear combination of few basis functions (Kaiser, 1958; Kayser and Tenke, 2003; Richman, 1986).
(4) *Rearrangement of PC vectors to TFDs*. The three PCs that explained the maximum variance in the matrix were selected, and re-arranged into three-dimensional matrices (i.e., with the same number of dimensions of the original single-trial TFDs). Note that the number of PCs was chosen empirically based on our previous studies (Iannetti et al., 2008; Mayhew et al., 2010; Mouraux et al., 2003), where three main PCs represented the stimulus-elicited ERP, ERD and ERS. The obtained PCs were then re-arranged from K_P_ × N_TF_ (two dimensions: PC numbers × time-frequency points) to K_P_ × (N_T_ × N_F_) (three dimensions: PC numbers × time points × frequency points).
(5) *TFD thresholding*. To isolate significant signal changes from background noise, the time-frequency maps of each PC were thresholded using a cut-off at two standard deviations from the mean of all time-frequency points (Mayhew et al., 2010). The effect of TFD thresholding using different cut-off levels on the performance of single-trial estimation is shown in the Supplementary Materials. Therefore, only the time-frequency points with amplitudes above (ERP and ERS) or below (ERD) two standard deviations from the mean were retained. The value of all other time-frequency points was set to zero (Mayhew et al., 2010).

**Fig. 2:**
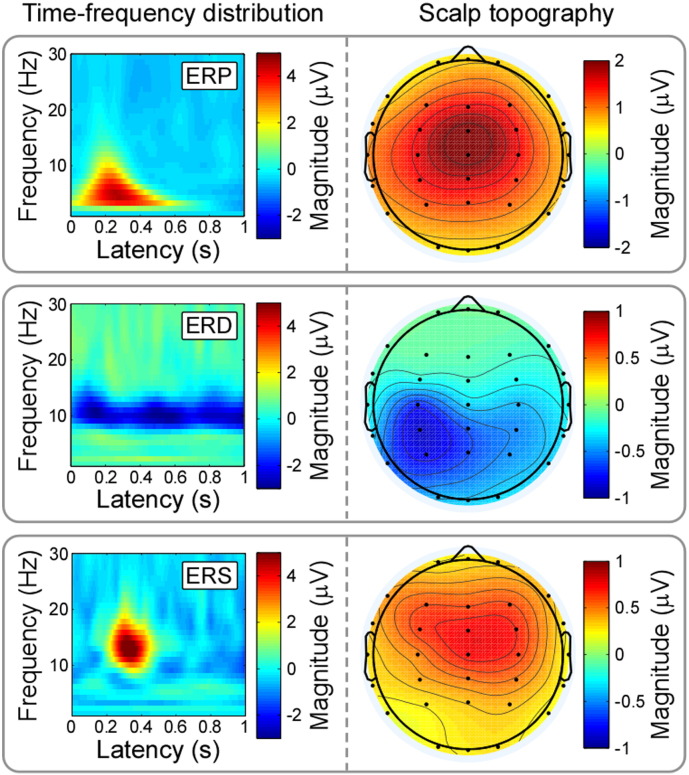
Time-frequency distributions of ERP/ERD/ERS and their scalp topographies. Group-level TFDs and scalp topographies of ERP, ERD, and ERS responses elicited by laser stimulation of the right hand dorsum. The scalp topography of the ERP response is centrally distributed and maximal at the vertex, similarly to that of the N2–P2 complex in the time domain (top panel). The scalp topography of the ERD response has a maximum contralateral to the stimulated side (middle panel). The scalp topography of the ERS response is symmetrically distributed, with a maximum on the frontal electrodes (bottom panel). These different topographies suggest that the three TFD responses have different underlying neural sources.

The scalp topography of each thresholded TFD was computed by spline interpolation of the mean power of the 20% time-frequency points displaying the highest power increase (ERP and ERS) or decrease (ERD), across all channels (Fig. 2). This “top 20%” summary value reflects, in each subject/trial, the highest response magnitudes in the thresholded TFD for each TF-feature, and reduces the noise that would be presented by including in the mean all points of the spectrogram, some of which would display little or no response. This approach avoids only selecting outlier values, and it has been successfully applied in previous studies (Iannetti et al., 2008; Iannetti et al., 2005b; Mayhew et al., 2010; Mitsis et al., 2008; Mouraux and Iannetti, 2008).

### EEG data analysis: single-trial analysis

**Fig. 3:**
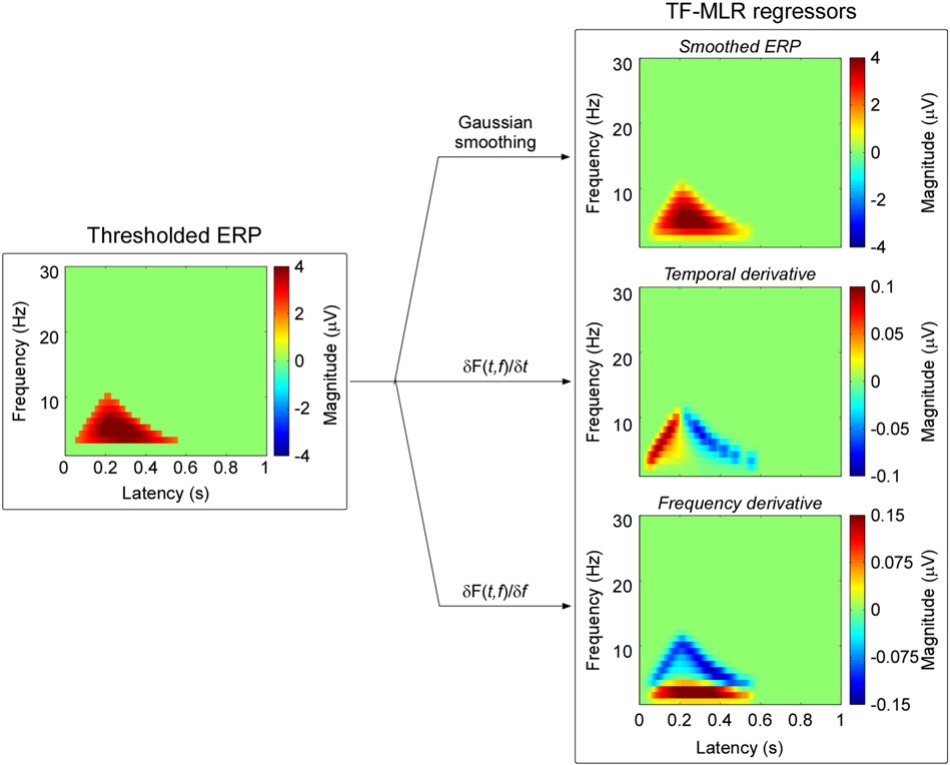
Generation of regressors in the TF-MLR approach. Left panel: Time-frequency representation of one of the thresholded features (‘ERP’, in this example) of the EEG response, obtained by PCA decomposition with Varimax rotation (Fig. 1). Right Panel: The three regressors obtained by the TF-MLR approach represent the average (Gaussian smoothing; the spread parameters of the Gaussian kernel are: σ_x_ = 30 ms, σ_y_ = 3 Hz), the temporal derivative and the frequency derivative of the ERP response, respectively. The temporal and frequency derivatives will be used to capture the variability in latency and frequency of single-trial TFDs.

**Fig. 4:**
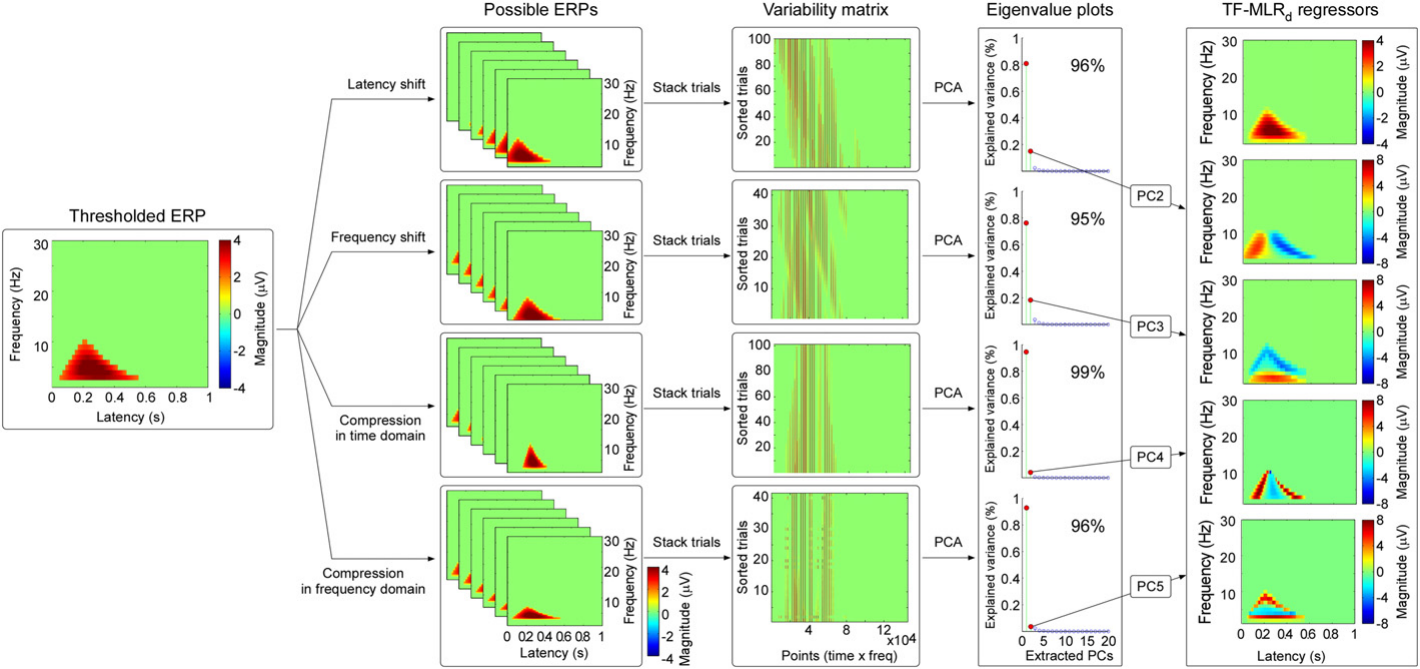
Generation of regressors in the TF-MLR_d_ approach. First column: Time-frequency representation of one of the thresholded features (‘ERP’, in this example) of the EEG response, obtained by PCA decomposition with Varimax rotation (see Fig. 1). Second column: TFDs representing plausible variability in latency, frequency, morphology in the time domain, and morphology in the frequency domain were generated by shifting and compressing the thresholded response in an enumerative fashion. Third column: For each source of variability, the plausible responses were re-arranged into vectors, which were subsequently stacked into a data matrix (variability matrix). Fourth column: The eigenvalue plots show the explained variance for each of the first 20 generated PCs, for each variability matrix. Note that the first two PCs explain the largest part of the total variance of each source of variability. Fifth column: Five regressors capturing the average magnitude of the considered TF-feature, and its variability in latency (the second PC in latency-shift variability matrix), in frequency (the second PC in frequency-shift variability matrix), in morphology in the time domain (the second PC in latency-compression variability matrix), and in morphology in the frequency domain (the second PC in frequency-compression variability matrix).

We describe two methods to estimate automatically the single-trial latency, frequency and magnitude of each time-frequency feature (ERP, ERD, and ERS): multiple linear regression in the time-frequency domain without a dispersion term (TF-MLR) and with a dispersion term (TF-MLR_d_). These methods are summarized in Figs. 3 and 4 respectively. Both methods have been developed into user-friendly software running under the MATLAB environment, which can be freely downloaded from www.iannettilab.net.

#### Time-frequency multiple linear regression (TF-MLR)

The MLR method to estimate the single-trial latency and amplitude of ERPs in the time domain was first described in Mayhew et al (2006), and successfully applied to the single-trial detection of the N1 wave of laser-evoked potentials (LEPs) (Hu et al., 2010), and of auditory-evoked potentials (AEPs) collected during simultaneous EEG-fMRI recording (Mayhew et al., 2010).

When extended in the time-frequency domain, such time-frequency MLR (TF-MLR) approach takes into account not only the latency jitter of the examined TF-feature, but also its variability in frequency. Thus, the variability in single trials can be modelled as follows.

(2) $$\begin{array}{c} F\left(t,f\right)=k_{1}F_{1}\left[\left(t+a_{1}\right),\left(f+b_{1}\right)\right]+k_{2}F_{2}\left[\left(t+a_{2}\right),\left(f+b_{2}\right)\right]\\ +k_{3}F_{3}\left[\left(t+a_{3}\right),\left(f+b_{3}\right)\right]+\varepsilon \end{array}$$

Where *F*(*t*,*f*) is the TFD of a single-trial LEP waveform which represents as a joint function of time *t* and frequency *f*, and *F*_1_(*t*,*f*), *F*_2_(*t*,*f*), and *F*_3_(*t*,*f*) are the across-trial averages of ERP, ERD, and ERS, respectively. *F*(*t*,*f*) can be modelled as the weighted sum of the ERP, ERD, and ERS, plus background noise ε. As unknown parameters in the single-trial analysis, *k*_1_, *k*_2_ and *k*_3_ are the weighted constants; *a*_1_, *a*_2_ and *a*_3_ are the values representing the variability in latency; and *b*_1_, *b*_2_ and *b*_3_ are the values representing the variability in frequency of ERP, ERD and ERS respectively.

Using the Taylor expansion, the MLR model can be simplified as:

(3) $$\begin{array}{c} F\left(t,f\right)\approx k_{1}F_{1}\left(t,f\right)+k_{1}a_{1}\frac{\partial F_{1}\left(t,f\right)}{\partial t}+k_{1}b_{1}\frac{\partial F_{1}\left(t,f\right)}{\partial f}\\ +k_{2}F_{2}\left(t,f\right)+k_{2}a_{2}\frac{\partial F_{2}\left(t,f\right)}{\partial t}+k_{2}b_{2}\frac{\partial F_{2}\left(t,f\right)}{\partial f}\\ +k_{3}F_{3}\left(t,f\right)+k_{3}a_{3}\frac{\partial F_{3}\left(t,f\right)}{\partial t}+k_{3}b_{3}\frac{\partial F_{3}\left(t,f\right)}{\partial f} \end{array}$$

where $\frac{\partial F_{1}\left(t,f\right)}{\partial t}$, $\frac{\partial F_{2}\left(t,f\right)}{\partial t}$, and $\frac{\partial F_{3}\left(t,f\right)}{\partial t}$ are the temporal derivatives of ERP, ERD, and ERS; and $\frac{\partial F_{1}\left(t,f\right)}{\partial f}$, $\frac{\partial F_{2}\left(t,f\right)}{\partial f}$, and $\frac{\partial F_{3}\left(t,f\right)}{\partial f}$ are the frequency derivatives of ERP, ERD, and ERS respectively. Thus, a single-trial TFD can be approximated as the sum of a set of weighted basis (average, its temporal derivative and its frequency derivative) (Fig. 3).

**Fig. 5:**
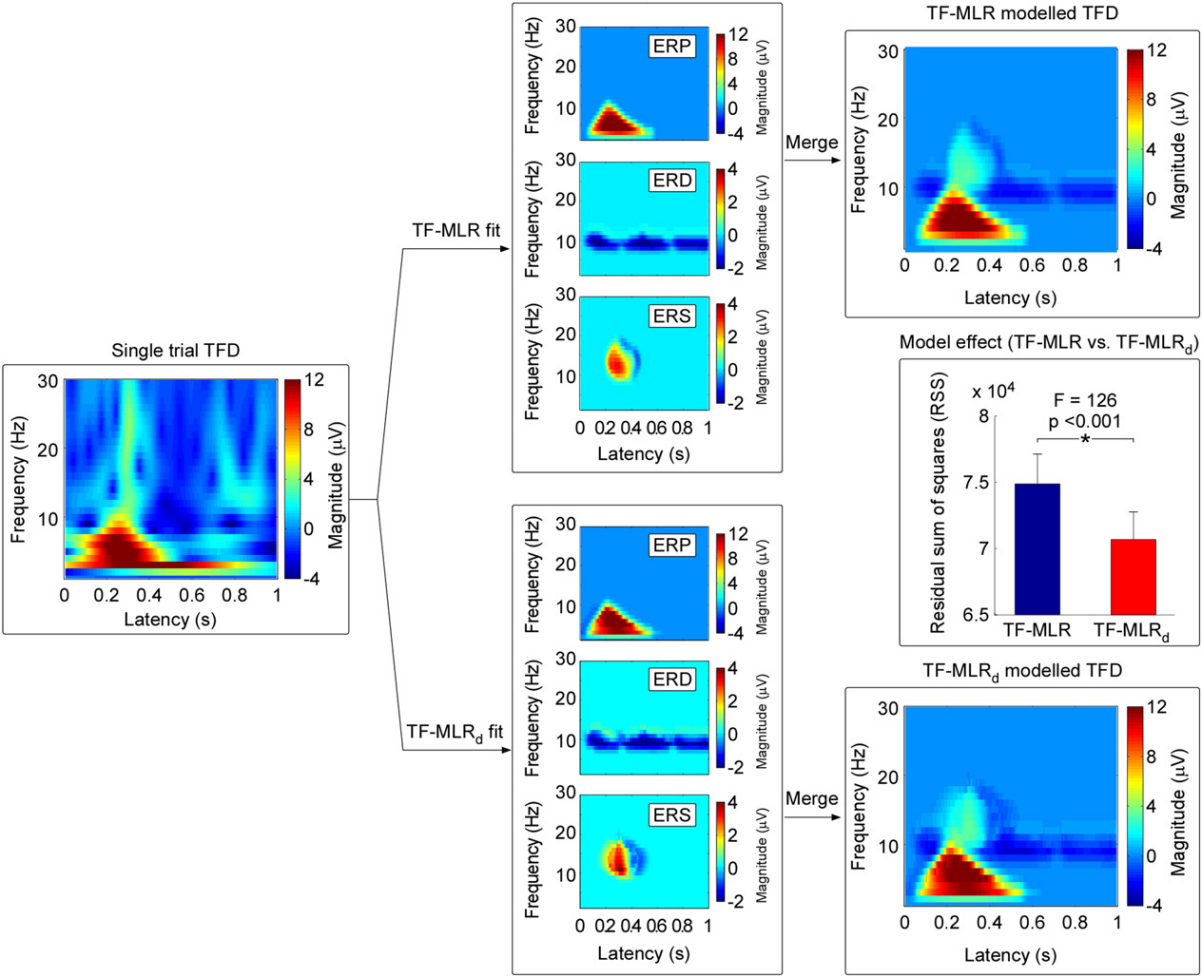
Example of a single-trial TFD modelled using both TF-MLR and TF-MLR_d_. Left panel: A single-trial TFD of a laser-elicited EEG response recorded at electrode Cz. Middle panel: Time-frequency response features (ERP, ERD and ERS) fitted using TF-MLR and TF-MLR_d_. Note that the information-of-interest (i.e., the ERP, ERD and ERS responses) is preserved, while the information-of-no-interest (e.g., background EEG noise) is largely removed, thus increasing the response SNR. Right panel: The sum of the fitted ERP, ERD and ERS responses constituted the modelled single-trial TFD. TF-MLR_d_ provides a significantly better fit of single trials than TF-MLR (*F* = 126, p < 0.001).

Based on the fitted single-trial TFD (Fig. 5, top panel), we calculated, for each TF-feature, the correlation coefficient (*CC*_1_, *CC*_2_ and *CC*_3_ for ERP, ERD and ERS, respectively) between the fitted single-trial TFD and the thresholded TFD obtained from PCA decomposition with Varimax rotation. Single-trial ERP and ERS magnitudes were finally obtained by calculating the mean of the 20% of points (relative to all the non-zero points in the thresholded TFD for each TF-feature [Fig. 1, right panel], the same hereinafter) displaying the highest increase (if *CC*_1_ > 0 or *CC*_3_ > 0, i.e., a positive fit) or the highest decrease (if *CC*_1_ < 0 or *CC*_3_ < 0, i.e., a negative fit), respectively. In contrast, single-trial ERD magnitude was obtained by calculating the mean of the 20% of points displaying the highest decrease (if *CC*_2_ > 0, i.e., a positive fit), or the highest increase (if *CC*_2_ < 0, i.e., a negative fit). Finally, single-trial latencies and frequencies corresponding to the measured ERP/ERD/ERS were obtained by calculating the mean latency and frequency of the selected “top 20%” of points in the time-frequency plane.

#### TF-MLR with dispersion term (TF-MLR_d_)

Similarly to what observed in ERP waveforms (Mouraux and Iannetti, 2008; Spencer, 2005), the time-frequency response averaged across trials is more dispersed in both time and frequency domains compared to each of single-trial TFDs, because of the latency and frequency variations from trial to trial.

To obtain an accurate estimate of single trial time-frequency responses, not only their variability in latency and frequency, but also their variability in morphology (both in time and frequency domain) should be taken into account. This has a physiological rationale. For example, in some clinical conditions (e.g., optic neuritis during multiple sclerosis), visual-evoked potentials are “desynchronized”, i.e., their amplitudes are reduced because of increased latency jitter, as well as increased width of single-trial responses (Orssaud, 2003; Pelosi et al., 1997). Latency jitter, as well as trial-by-trial variability in response morphology (i.e., wave width or frequency variability) could thus be important parameters for clinical studies. Therefore, in addition to the basis set of TF-MLR, two more regressors, representing the scaling of the single-trial response in time or frequency domain are considered, which leads to the following TF-MLR_d_ model (Fig. 4):

(4) $$\begin{array}{c} F\left(t,f\right)=k_{1}F_{1}\left[\left(s_{1}t+a_{1}\right),\left(c_{1}f+b_{1}\right)\right]+k_{2}F_{2}\left[\left(s_{2}t+a_{2}\right),\left(c_{1}f+b_{2}\right)\right]\\ \;+k_{3}F_{3}\left[\left(s_{3}t+a_{3}\right),\left(c_{1}f+b_{3}\right)\right]+\varepsilon \; \end{array}$$

where *s*_1_, *s*_2_ and *s*_3_ are the coefficients that determine the compression ratios of the time width of ERP, ERD and ERS of each single-trial TFD compared to those of the average TFD, respectively, while *c_1_*, *c*_2_ and *c*_3_ are the coefficients that determine the compression ratios of the frequency width of ERP, ERD and ERS of each single-trial TFD compared to those of the average TFD, respectively.

To estimate the unknown parameters *k*, *s*, *c*, *a*, and *b* of each single-trial TFD, we generate a basis set to fit the ERP, ERD and ERS by employing PCA, i.e., a non-parametric data-driven approach (Jolliffe, 2002). We generated five PCs representing, for each feature of the TFD (1) the average of the response, (2) the variability in latency, (3) the variability in frequency, (4) the variability in morphology in the time domain, and (5) the variability in morphology in the frequency domain. The procedure for generating the TF-MLR_d_ regressors consists in the following five steps (Fig. 4).

(1) *Generating the variability matrices* (Fig. 4, step 1). For each TF-feature, four groups of plausible TFDs were generated by shifting ([1] from − 100 ms to 100 ms in steps of 2 ms in the time domain, [2] from − 2 Hz to 2 Hz in steps of 0.1 Hz in the frequency domain, centred at the peak of the TF-feature) and compressing ([3] from 1 to 2 in steps of 0.01 in the time domain, [4] from 1 to 2 in steps of 0.025 in the frequency domain, centred at the peak of the TF-feature) the thresholded TFD in an enumerative fashion using resampling technique (i.e., increasing the frequency resolution before shifting and compressing the plausible TFDs, as well as decreasing the frequency resolution afterwards). Each of these plausible bidimensional TFDs was re-arranged into a monodimensional vector, and all vectors were stacked to form four bidimensional data matrices that we called variability matrices (latency-shift matrix, frequency-shift matrix, latency-compression matrix, frequency-compression matrix), for each TF-feature. Note that the variability of TF-feature magnitude is captured by the coefficients weighting the basis sets, and thus it is not included in these variability matrices.
(2) *PCA separation* (Fig. 4, step 2). Each of the four variability matrices was separately fed into a PCA, to obtain the PCs representing the linear subspace for each variability matrix. As the first few PCs are responsible for most of the data variance (Hossein-Zadeh et al., 2003; Jolliffe, 2002), it is expected that the first PCs resulting from the variability matrices would represent the average TFD for each TF-feature and the second PCs embody its variability in latency, frequency, morphology in the time domain, and morphology in the frequency domain.
(3) *Basis set definition* (Fig. 4, step 3). Since the second PCs resulting from the variability matrices always captured the variability of latency, the variability of frequency, the variability of morphology in the time domain, and the variability of morphology in the frequency domain, the basis sets (five regressors) for each TF-feature was defined by [1] the average of each thresholded time-frequency features (Gaussian smoothed, Mayhew et al., 2010); [2] the second PC from the latency-shift variability matrix; [3] the second PC from the frequency-shift variability matrix; [4] the second PC from the latency-compression variability matrix; [5] the second PC from the frequency-compression variability matrix.
(4) *Single-trial fitting* (Fig. 5, bottom panel). The obtained basis sets were regressed against the TFD of each single trial. The coefficient (i.e., β value) of each of the five regressors was estimated by the least square approach, as described in Mayhew et al (2006) and Hu et al (2010). By multiplying these estimated coefficients by the corresponding regressors, the fitted TFDs of each single trial were reconstructed.
(5) *Single-trial latency, frequency, and magnitude estimation*. Based on the fitted single-trial TFD (Fig. 5, bottom panel), we calculated the correlation coefficients (*CC*_1_, *CC*_2_, and *CC*_3_ for ERP, ERD, and ERS respectively) between the fitted single-trial TFD and the threshold TFD obtained from PCA decomposition with Varimax rotation for each TF-feature. The calculation of single-trial parameters is the same as the approach described for TF-MLR.

### Single-trial performance assessment

The performance of the TF-MLR and TF-MLR_d_ in estimating single-trial parameters was assessed both on simulated and real EEG datasets (performance on simulated data is reported in the Supplementary Materials).

(1) *Goodness of fit (TF-MLR vs. TF-MLR_d_)*. Compared with the TF-MLR approach, the TF-MLR_d_ takes the variability of morphology (both latency and frequency) into consideration, and thus generates two additional basis sets for each TF-feature. To determine whether the TF-MLR_d_ approach gives a significantly better fit to the data than the TF-MLR approach regardless of the number of basis sets in each of the two approaches, we performed an *F* test, which takes into account the number of the compared model parameters (Motulsky and Christopoulos, 2004). Let's consider two models, 1 and 2, where model 1 is “nested” within model 2. That is, model 1 has *p*_1_ parameters, and model 2 has *p*_2_ parameters, where *p*_2_ > *p*_1_, and, for any choice of parameters in model 1, the same regression curve can be fitted by some choice of the parameters in model 2. It is obvious that in this example the model with more parameters will always fit the data at least as well as the model with fewer parameters. Therefore, any method to compare a simple model with a more complicated model has to balance the decrease in sum-of-squares with the increase in the number of parameters. This can be achieved using the *F* test (Eq. (5)), which determines whether model 2 gives a significantly better fit to the data than model 1 regardless of the number of parameters (Motulsky and Christopoulos, 2004), as follows:
  (5) $$F=\frac{\left({RSS}_{1}-{RSS}_{2}\right)/\left(p_{2}-p_{1}\right)}{{RSS}_{2}/\left(n-p_{2}\right)}$$
  where RSS_1_ and RSS_2_ are the residual sum-of-squares of model 1 and model 2 respectively, and *n* is the number of data points.
  *F* will have an *F* distribution with (*p*_2_ − *p*_1_, *n* − *p*_2_) degrees of freedom. The null hypothesis is that model 2 does not provide a significantly better fit than model 1, and this hypothesis should be rejected if the *F* value is greater than the critical value of the *F* distribution for a desired false-rejection probability (p < 0.05).
(2) *Detection bias*. To examine whether the two explored approaches (TF-MLR and TF-MLR_d_) introduce a biase into the analysis by, for example, fitting noise, each of them was applied to resting EEG epochs obtained from the same subjects. Such possible detection bias was quantified by comparing the obtained magnitudes of each TF-feature against zero, using a one sample *t* test.
(3) *Comparison of single-trial magnitudes (TF-MLR vs. TF-MLR_d_)*. Single-trial magnitudes of each TF-feature obtained using the TF-MLR approach were averaged across trials and compared to the corresponding values obtained using the TF-MLR_d_ approach. Their differences were then assessed using a paired sample *t* test.
(4) *Correlation between different single-trial estimates, as well as correlation between single-trial estimates and corresponding single-trial subjective pain intensity*. Single-trial parameters (latency, frequency, and magnitude) obtained using the TF-MLR and TF-MLR_d_ approaches were further compared with each other, as well as with the single-trial ratings of the subjective pain intensity. For both approaches, all possible correlations (between each estimated single-trial parameter and the corresponding subjective pain intensity, and between each pair of the estimated single-trial parameters) were performed for each subject (Iannetti et al., 2005a). The obtained correlation coefficients were transformed to Z values using Fisher R-to-Z transformation. The resulting Z values were finally compared against zero using a one sample *t* test.

## Results

### EEG data analysis: TF-feature separation

Fig. 1 shows the first three PCs obtained from PCA decomposition with Varimax rotation. These three PCs explained the largest amount of variance (23.1%, 9.2% and 5.9%, respectively), while the explained variance for any of the remaining PCs was less than 5%.

After thresholding using the two-SD cut-off, the amount of background EEG noise on the time-frequency plane was remarkably reduced while the regions corresponding to ERP/ERD/ERS were clearly preserved (Fig. 1). The ERP region (feature 1) was located at 50–550 ms post-stimulus (in time) and 3–10 Hz (in frequency); the ERD region (feature 2) was located at 50–1000 ms and 9–12 Hz; and the ERS region (feature 3) was located at 217–447 ms and 10–19 Hz. The group-level PLVs indicated that only the ERP response was phase-locked to stimulus onset, while the other TFD responses (ERD and ERS) were not (Fig. 1, left panel). Similar time-frequency distributions of the EEG responses elicited by transient laser pulses (Iannetti et al., 2008; Mouraux et al., 2003; Mouraux and Iannetti, 2008; Ohara et al., 2004; Ploner et al., 2006) and by transient auditory stimuli (Makinen et al., 2004; Mayhew et al., 2010) have been reported previously.

Fig. 2 shows the scalp topographies of the ERP, ERD, and ERS responses elicited by laser stimulation of the right hand dorsum. While the scalp topography of ERP response was centrally distributed and maximal at the vertex, the scalp topography of ERS was slightly more frontal. The scalp topography of the ERD response had a maximum contralateral to the stimulated side, and for this reason single-trial ERD parameters were measured from electrode C3. The similarity between the scalp topography of ERP in the time-frequency domain and the scalp topography of N2–P2 complex in the time domain (Mouraux and Iannetti, 2009), together with the observation of their similar modulation by different experimental factors (Iannetti et al., 2008; Schulz et al., 2011; Valentini et al., 2011; Zhang et al., 2012), suggests that the neural activity reflected in the N2–P2 complex in the time domain corresponds to the ERP region in the time-frequency domain.

### Single-trial performance assessment

Fig. 3 shows the three regressors for the ERP response (i.e., the thresholded feature 1) generated using the MLR approach. These regressors represent the average amplitude (Gaussian smoothed) of the response, its temporal derivative and its frequency derivative. The regressors for the ERS and ERD responses were generated in the same way.

Fig. 4 shows the procedure used to generate the regressors for the ERP response (thresholded feature 1), in the TF-MLR_d_ approach. The first two PCs, obtained from the latency-shift, frequency-shift, latency-compression, and frequency-compression variability matrices, explained 96%, 95%, 99%, and 96% of the total variance, respectively. The same procedure was used to generate regressors for the ERS and ERD responses.

When comparing the regressors obtained using the TF-MLR and TF-MLR_d_ approaches (Figs. 3 and 4), we observed that the temporal derivative in TF-MLR is very similar to the second PC of the latency-shift variability matrix in TF-MLR_d_, and that the frequency derivative in TF-MLR is very similar to the second PC of the frequency-shift variability matrix in TF-MLR_d_. In addition, the second PC obtained from the latency-compression variability matrix and the second PC obtained from the frequency-compression variability matrix were included in the TF-MLR_d_ approach. Thus, the TF-MLR_d_ analysis can better explain the variability of single-trial TFD than the TF-MLR analysis, especially for the variability of response morphology (Hu et al., 2011a), both in the time domain and in the frequency domain.

**Fig. 6:**
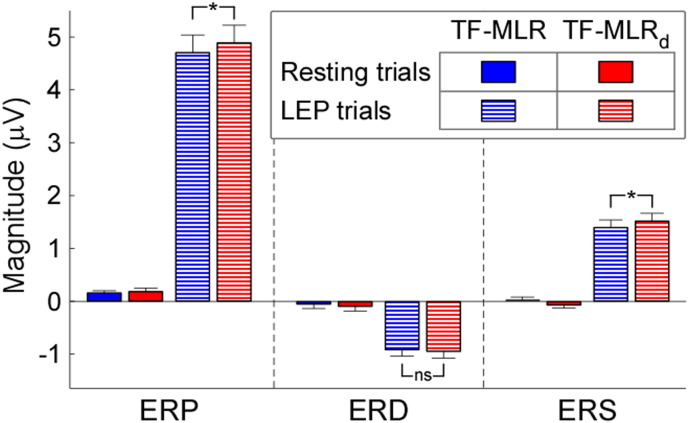
Comparison of single-trial magnitudes estimated using TF-MLR and TF-MLR_d_ of LEP trials and resting EEG trials. Single-trial magnitudes of ERP (left), ERD (middle), and ERS (right) estimated using TF-MLR (blue) and TF-MLR_d_ (red) from LEP trials (palisaded) and resting EEG trials (filled). For both TF-MLR and TF-MLR_d_, the magnitudes of ERP, ERD and ERS estimated from resting EEG trials are not significantly different from zero (p > 0.05 for all comparisons; one sample *t* test). Whereas the single-trial magnitudes of ERD estimated from LEP trials using TF-MLR and TF-MLR_d_ are not significantly different, the single-trial magnitudes of ERP and ERS estimated from LEP trials using TF-MLR were significantly smaller than those estimated using TF-MLR_d_ (p < 0.01 for both comparisons, paired sample *t* test).

**Fig. 7:**
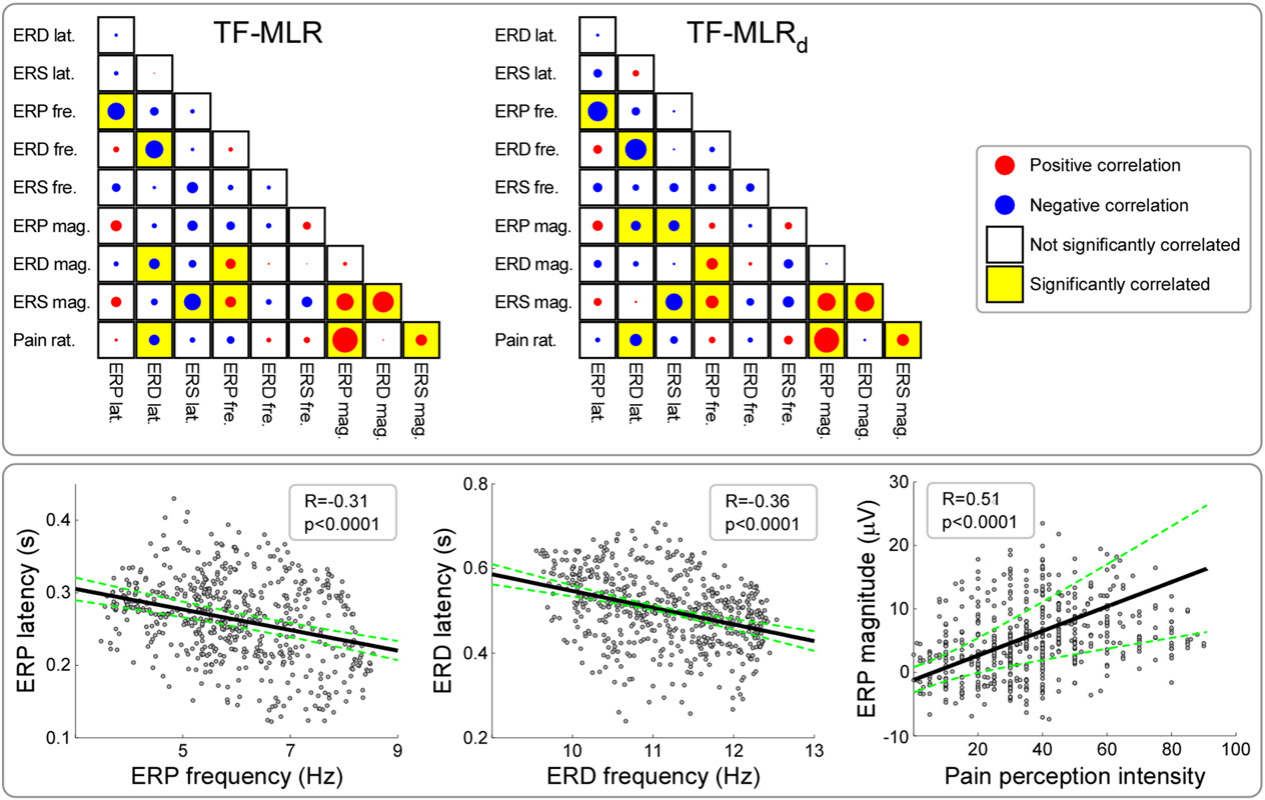
Correlation between different single-trial estimates, as well as correlation between single-trial estimates and the corresponding single-trial subjective pain intensity. Top panel: All possible correlations between different single-trial estimates, measured using TF-MLR (left) and TF-MLR_d_ (right), as well as between these single-trial estimates and the corresponding subjective pain intensity. Red and blue dots represent positive and negative correlations. When a correlation was significant, the corresponding box was marked in yellow. Note that the correlations were markedly similar between TF-MLR and TF-MLR_d_. Bottom panel: Representative correlations between different single-trial estimates, measured using TF-MLR_d_. The single-trial ERP latencies showed a significant negative correlation with the corresponding ERP frequencies (mean R = − 0.31 ± 0.07, p < 0.0001; left). The single-trial ERD latencies showed a significant negative correlation with the corresponding ERD frequencies (mean R = − 0.36 ± 0.09, p < 0.0001; middle). The single-trial ERP magnitudes showed a significant positive correlation with the corresponding pain perception intensity (mean R = 0.51 ± 0.15, p < 0.0001; right).

(1) *Goodness of fit assessment (TF-MLR vs. TF-MLR_d_)*. The top and bottom panels of Fig. 5 show the fitted responses of a same single-trial using TF-MLR and TF-MLR_d_, respectively. In the fitted response the information-of-interest, reflected in the ERP, ERS and ERD, was correctly preserved, while the information-of-no-interest, represented by the stimulus-unrelated background EEG was removed. This procedure increased the SNR of the brain responses (both phase-locked and non-phase-locked) in the time-frequency plane. Importantly, the *F* test performed between the goodness of fit obtained with the TF-MLR and the TF-MLR_d_ approaches indicated that the better performance of TF-MLR_d_ was not simply due to the larger number of regressors (*F* = 126, p < 0.001; Fig. 5, right panel), but to the actual fitting of physiologically-relevant sources of variability (e.g., variability of morphology in the time and frequency domains).
(2) *Detection bias*. To test whether the methods (TF-MLR and TF-MLR_d_) used to estimate single-trial magnitude of ERP, ERS and ERD introduced any detection bias, they were applied to an equal number of resting EEG epochs obtained from all subjects. When estimated using TF-MLR approach, the mean (± SEM) of single-trial estimate of response magnitude in resting EEG epochs were 0.15 ± 0.12 μV, − 0.09 ± 0.21 μV, and − 0.06 ± 0.14 μV for ERP, ERD, and ERS. These magnitude values were not significantly different from zero (ERP: p = 0.24; ERD: p = 0.69; ERS: p = 0.68, one sample *t* test). When estimated using the TF-MLR_d_ approach, the mean (± SEM) single-trial estimate of response magnitude in resting EEG epochs were 0.19 ± 0.14 μV, − 0.04 ± 0.20 μV, and 0.001 ± 0.14 μV for ERP, ERD, and ERS. These magnitude values were not significantly different from zero (ERP: p = 0.22; ERD: p = 0.83; ERS: p = 0.99, one sample *t* test). These results clearly show that both TF-MLR and TF-MLR_d_ approaches provide an unbiased estimate of single-trial magnitude of ERP, ERS and ERD. A comparison of the single-trial magnitude values obtained from LEP trials vs. the resting EEG trials using the two approaches is shown in Fig. 6.
(3) *Comparison of single-trial magnitudes (TF-MLR vs. TF-MLR_d_)*. Fig. 6 shows the comparison of the average of single-trial ERP, ERD and ERS magnitudes estimated using TF-MLR and TF-MLR_d_. The single-trial ERP and ERS magnitudes estimated using TF-MLR were significantly smaller than those estimated by TF-MLR_d_ (ERP: 4.70 ± 0.79 μV vs. 4.93 ± 0.80 μV; p < 0.007; ERS: 1.39 ± 0.35 μV vs. 1.52 ± 0.35 μV; p < 0.008, paired sample *t* test). In contrast, there was no significant difference between the single-trial ERD magnitudes estimated using TF-MLR and TF-MLR_d_ (ERD: − 0.91 ± 0.29 μV vs. − 0.94 ± 0.32 μV; p > 0.05, paired sample *t* test).
(4) *Correlation between different single-trial estimates, as well as correlation between single-trial estimates and corresponding single-trial subjective pain intensity.* Fig. 7 shows all possible correlations between single-trial parameters estimated using TF-MLR and TF-MLR_d_. Overall, correlations were markedly similar in the data estimated using TF-MLR and TF-MLR_d_. We observed significant negative correlations between single-trial ERP latencies and the corresponding ERP frequencies (mean R = − 0.31 ± 0.07, p < 0.0001, obtained from TF-MLR_d_, hereinafter), and between the single-trial ERD latencies and the corresponding ERD frequencies (mean R = − 0.36 ± 0.09, p < 0.0001). In addition, we observed significant positive correlations between the single-trial ERP and ERS magnitudes and the corresponding subjective pain intensity (ERP: mean R = 0.51 ± 0.15, p < 0.0001; ERS: mean R = 0.15 ± 0.19, p = 0.03).

## Discussion

The present study shows that different features (i.e., ERP, ERD, and ERS) of the time-frequency EEG response elicited by transient sensory stimuli can be (1) isolated and characterized using PCA with Varimax rotation, and (2) reliably estimated at single-trial level using multiple linear regression approaches (TF-MLR and TF-MLR_d_). Such approaches allow estimating the trial-to-trial variability of the latency, frequency and magnitude of several time-frequency features. When testing such approaches on a real EEG dataset we observed meaningful correlations between different stimulus parameters and the subjective sensations elicited by a somatosensory stimulus.

### PCA-separation of different time-frequency features

We showed that PCA with Varimax rotation can extract successfully different time-frequency features of the EEG response elicited by a transient stimulus (Fig. 1). Such approach has been previously applied to separate and quantify different ERP peaks in the time domain (Dien, 2010; Dien et al., 2007; Kayser and Tenke, 2003), as well as different features of the time-frequency EEG responses (Bernat et al., 2007; Bernat et al., 2005; Mayhew et al., 2010). It should be noted that PCA converts a set of observations of possibly correlated variables into a set of principal components, which are orthogonal (i.e., linearly uncorrelated; Jolliffe, 2002). This orthogonality is still present after the Varimax rotation, which is normally applied to the PCA solution to simplify the structure of the components by maximizing the sum of the variances of the squared loadings (Kaiser, 1958). Dien (1998) argued that the orthogonality requirement of PCA with Varimax rotation (i.e., the requirement that the components should be linearly uncorrelated) may constitute a problem when extracting ERP components, given their possible intrinsic correlation. To address this issue, Dien (1998) suggested the use of oblique/non-orthogonal rotations (e.g., Promax rotation), since these non-orthogonal rotations relax the requirement of orthogonality of the Varimax rotation. However, it should be also noted that the requirement of non-orthogonality has been suggested to represent a disadvantage *per se* (Kayser and Tenke, 2003), since non-orthogonal components are dependent on each other, resulting in the increase of the probability of Type I errors in subsequent statistical analyses (Kayser and Tenke, 2003).

It should be also noted that to minimize overinterpretations and misindentifications, researchers have been suggested to examine carefully the time/time-frequency distribution, scalp topography, and sensitivity to experimental manipulations of the PCA-isolated components, based on a priori knowledge of well-characterized event-related features (Fabiani et al., 1987). Following this suggestion, we have closely compared the components isolated by PCA with Varimax rotation with previously-documented time-frequency features in LEP studies. Several previous studies (Iannetti et al., 2008; Mouraux et al., 2003; Ohara et al., 2004; Ploner et al., 2006; Stancak et al., 2003) consistently reported that laser-evoked EEG responses show three typical time-frequency features: a phase-locked ERP, and a non-phase-locked ERD and ERS (Fig. 1). Importantly, these three time-frequency features are at least partially independent, as demonstrated by their differential sensitivity to experimental manipulations (Iannetti et al., 2008; Mouraux et al., 2003). The phase-locked response (ERP: 50–550 ms, 3–10 Hz), mainly located in theta band, corresponds to the time-frequency representation of the N2–P2 complex of LEPs in the time domain (Mouraux et al., 2003). Indeed, the scalp topography of the ERP response is similar to that of the N2–P2 complex, i.e., maximal at the vertex and symmetrically distributed over both hemispheres (Fig. 2). Therefore, the time-frequency ERP response in the theta band is likely to reflect neural activities from the anterior cingulate cortex (ACC) and bilateral operculo-insular areas, which are regarded as the main generators of the N2–P2 complex of the LEPs (Frot et al., 2008; Garcia-Larrea et al., 2003). The non-phase-locked ERD in the alpha band (50–1000 ms, 9–12 Hz) was maximal over the parietal region contralateral to the stimulated hand (Fig. 2). This feature might reflect activation/disinhibition of the primary sensorimotor cortex (Hu et al., 2013; Neuper and Klimesch, 2006; Pfurtscheller and Neuper, 1994). In addition, the non-phase-locked ERS (217–447 ms, 10–19 Hz), mainly located in the beta band, was symmetrically distributed, with a maximum over the frontal regions (Fig. 2). This feature may partly reflect neural activities in the prefrontal cortex (PFC), which is commonly activated in response to nociceptive stimuli in most fMRI studies, but rarely reported in time domain EEG/MEG studies (Apkarian et al., 2005). These observations suggest the presence of a neurovascular coupling, resulting in a Blood Oxygenation Level Dependent (BOLD) signal measured by fMRI, not only triggered by the phase-locked responses measured by EEG/MEG in the time domain (Debener et al., 2005; Liu et al., 2012), but also by the non-phase-locked oscillatory responses (Iannetti and Mouraux, 2009).

It should be noted that different features of the EEG response (e.g., the ERP and ERS) overlap in both time and frequency, and have different time and frequency limits in different subjects. Previously, single-trial magnitudes of such responses have been simply calculated from the mean of the “top 20%” points within a given time-frequency region-of-interests (TF-ROI), empirically defined based on the group average time-frequency distributions (e.g., Iannetti and Mouraux, 2009; Mayhew et al., 2010). Therefore, this TF-ROI approach has two major limitations: (1) it cannot isolate different but overlapping time-frequency features, and (2) it ignores the between-subject variability of the TF-ROI limits.

PCA with Varimax rotation allows overcoming these two crucial limitations. Indeed, it can separate physiologically distinct EEG activities, even if they overlap in the time domain (Dien, 2010; Dien et al., 2007; Kayser and Tenke, 2003), in the time-frequency domain, or both (Fig. 1). Furthermore, when jointly applied with TF-MLR or TF-MLR_d_, it allows effectively modeling not only the within-subject, but also the between-subject variability of TF-ROI limits, using a set of regressors (e.g., the temporal derivative and the frequency derivative; Figs. 3–4).

We selected three PCs, which explained the maximum variance in single-trial TFDs and isolated the stimulus-evoked ERP, ERD and ERS. The number of selected PCs was determined empirically based on previous studies (Iannetti et al., 2008; Mayhew et al., 2010; Mouraux et al., 2003). When extending the same approach to other applications (e.g., stimulus-evoked EEG/MEG responses of other sensory modalities), it will be necessary to ensure that (1) the time-frequency features isolated by PCA are well studied, and (2) these features are clearly presented in the average TFDs. Also, the separation of time-frequency features using PCA with Varimax rotation could be, in principle, applied to single-trial TFDs measured at all recorded channels (thus yielding to four dimensions: trial number × time point × frequency point × channel). Although this approach will require significant computational resources,^1^ novel or more precise time–frequency features may be identified. Importantly, this technique may not be able to isolate some high-frequency features of the response (e.g., gamma band oscillations) due to their lower signal-to-noise ratio, as compared with the low frequency features (e.g., ERP, ERD, and ERS in the present study). To achieve this aim, the possibility of processing band-pass filtered TFDs (e.g., from 50 Hz to 100 Hz, when exploring gamma band oscillations) should be considered.

### Robust and unbiased single-trial estimate of time-frequency features

In the time domain, both MLR and MLR_d_ permit to estimate latency and amplitude of single-trial ERPs, in an automatic and unbiased fashion (Hu et al., 2011a; Hu et al., 2010; Mayhew et al., 2006). Here we described an important extension of these approaches, to allow estimating single-trial EEG responses in the time-frequency domain (TF-MLR and TF-MLR_d_; Figs. 3 & 4).

When comparing the goodness of fit obtained with the TF-MLR and TF-MLR_d_ approaches, the latter provides a significantly better fit to the single-trial time-frequency responses (*F* = 126, p < 0.0001) (Fig. 5, right panel). This observation indicates that the TF-MLR_d_ model fits the single-trial TFDs better than the TF-MLR model regardless of the number of basis sets employed in each of the two approaches. In addition, the magnitudes of single-trial ERP and ERS responses estimated by TF-MLR_d_ were significantly greater than those estimated by TF-MLR (ERP magnitude: 6.4 ± 7.3% increase, p < 0.007; ERS magnitude: 5.8 ± 19.4% increase, p < 0.008; two tailed *t* test) (Fig. 6). Importantly, both TF-MLR and TF-MLR_d_ approaches provide an unbiased estimate of single-trial magnitude of ERP, ERS and ERD (p > 0.05 for all comparisons, one sample *t* test) (Fig. 6). Indeed, when the stimulus does not elicit a physiological response (a condition we modelled using the resting EEG dataset), the average of the single-trial estimates of magnitude tends towards zero, under the unique assumption that a sufficiently high number of trials are estimated. In contrast, when estimated using the TF-ROI approach (estimate single-trial magnitudes from the mean of “top 20%” points within the pre-defined TF-ROIs, Mayhew et al., 2010), the mean (± SEM) of single-trial estimate of response magnitude in resting EEG epochs was always significantly different from zero (ERP: 2.74 ± 0.26 μV; ERD: − 2.81 ± 0.34 μV; ERS: 2.73 ± 0.30 μV; p < 0.001 for all comparisons, one sample *t* test).

Altogether, these findings indicate that the TF-MLR approach, which uses fewer regressors than the TF-MLR_d_ approach, is more *specific* when detecting stimulus-related responses (Friman et al., 2003), but is unable to capture the variability of the response morphology, both in the time domain and in the frequency domain. Therefore, the TF-MLR provides a simple and robust approach to estimate single-trial parameters, and is particularly appropriate for EEG responses with relatively low SNR (for example the early N1 wave of LEPs; Hu et al., 2010). In contrast, the higher number of regressors in the TF-MLR_d_ approach makes it more *sensitive* in detecting response variability (Hu et al., 2011a), with the possible drawback of fitting some noise (Friman et al., 2003). The TF-MLR_d_ approach is thus better suited to estimate accurately single-trial responses with relatively high SNR (e.g., intracranial recordings, interictal spikes in epilepsy patients). Therefore, TF-MLR and TF-MLR_d_ perform differently when applied to estimate single-trial time-frequency features with different SNR. This notion is also supported by the simulation study, in which we quantified the performance of the two approaches at controlled SNR levels (see Supplementary Materials). Lastly, given that it can be applied to any kind of trial-to-trial variability, the TF-MLR_d_ approach lends itself to a wide range of applications in system neuroscience, although the definition of the parameters to generate the variability matrices in TF-MLR_d_ (Fig. 4, step 1) could be improved using some prior knowledge of the possible range of the latency and frequency variability. In addition, the SNR can be significantly improved using spatial filtering (e.g., ICA and common spatial pattern analysis; Huang et al., 2013). Finally, multi-way analysis (e.g., tensor decomposition) of EEG data spanning the spatial-temporal-spectral domain could be considered to further enhance the SNR (Cichocki, 2013). After such filtering, TF-MLR and TF-MLR_d_ could be also used on independent components (ICs), or applied on spatially-filtered signals, thus obtaining single-trial estimates of brain responses with enhanced SNR.

Understanding the relationship between the magnitude of stimulus-evoked cortical responses and perceptual experiences is the objective of perceptual neuroscience (Mountcastle, 1998). Here we related the brain responses elicited by nociceptive specific laser stimuli (LEPs) with the corresponding painful percepts. Laser stimuli activate selectively free nerve endings of skin nociceptors (Iannetti et al., 2006) and LEPs are widely used to assess the function of nociceptive pathways in health and disease (Treede et al., 2003). In the time-frequency domain, we observed a strong correlation between the phase-locked ERP magnitude and subjective pain intensity (mean R = 0.51 ± 0.15, p < 0.0001; Fig. 7). This is in line with several previous studies (Carmon et al., 1978; Carmon et al., 1980; Iannetti et al., 2008; Kakigi et al., 1989), showing that the amplitude of the N2–P2 complex is often highly correlated with the subjective pain intensity. We also observed a positive correlation between non-phase-locked ERS magnitude and subjective pain intensity (mean R = 0.15 ± 0.19, p = 0.03).

In addition to exploring the trial-by-trial correlation between magnitude of the time-frequency EEG responses and perception, the described approaches provide a reliable estimation of other parameters defining an EEG response in the time-frequency domain. Besides response magnitude, these approaches allow estimating trial-by-trial variability in latency and frequency, as well as response dispersion in both time and frequency domains (Fig. 7). The availability of reliable estimates of all these parameters allows a more complete use of the information contained in stimulus-elicited EEG response, thus having the potential of providing novel physiological information in both basic and clinical studies.

## Footnotes

1 In the present study, approximately 6 GB of memory were needed to perform PCA separation with Varimax rotation on single-trial TFDs measured from a single channel.

## References

[bb0005] Apkarian A.V., Bushnell M.C., Treede R.D., Zubieta J.K. (2005). Human brain mechanisms of pain perception and regulation in health and disease. Eur. J. Pain.

[bb0015] Barbati G., Sigismondi R., Zappasodi F., Porcaro C., Graziadio S., Valente G., Balsi M., Rossini P.M., Tecchio F. (2006). Functional source separation from magnetoencephalographic signals. Hum. Brain Mapp..

[bb0010] Barbati G., Porcaro C., Hadjipapas A., Adjamian P., Pizzella V., Romani G.L., Seri S., Tecchio F., Barnes G.R. (2008). Functional source separation applied to induced visual gamma activity. Hum. Brain Mapp..

[bb0020] Baumgartner U., Cruccu G., Iannetti G.D., Treede R.D. (2005). Laser guns and hot plates. Pain.

[bb0030] Bernat E.M., Williams W.J., Gehring W.J. (2005). Decomposing ERP time-frequency energy using PCA. Clin. Neurophysiol..

[bb0025] Bernat E.M., Malone S.M., Williams W.J., Patrick C.J., Iacono W.G. (2007). Decomposing delta, theta, and alpha time-frequency ERP activity from a visual oddball task using PCA. Int. J. Psychophysiol..

[bb0035] Bingham E., Hyvarinen A. (2000). A fast fixed-point algorithm for independent component analysis of complex valued signals. Int. J. Neural Syst..

[bb0040] Bromm B., Treede R.D. (1984). Nerve fibre discharges, cerebral potentials and sensations induced by CO2 laser stimulation. Hum. Neurobiol..

[bb0045] Bromm B., Treede R.D. (1991). Laser-evoked cerebral potentials in the assessment of cutaneous pain sensitivity in normal subjects and patients. Rev. Neurol. (Paris).

[bb0060] Carmon A., Mor J., Goldberg J. (1976). Evoked cerebral responses to noxious thermal stimuli in humans. Exp. Brain Res..

[bb0050] Carmon A., Dotan Y., Sarne Y. (1978). Correlation of subjective pain experience with cerebral evoked responses to noxious thermal stimulations. Exp. Brain Res..

[bb0055] Carmon A., Friedman Y., Coger R., Kenton B. (1980). Single trial analysis of evoked potentials to noxious thermal stimulation in man. Pain.

[bb0065] Cichocki A. (2013). Tensor Decompositions: A New Concept in Brain Data Analysis?.

[bb0070] Cruccu G., Aminoff M.J., Curio G., Guerit J.M., Kakigi R., Mauguiere F., Rossini P.M., Treede R.D., Garcia-Larrea L. (2008). Recommendations for the clinical use of somatosensory-evoked potentials. Clin. Neurophysiol..

[bb0080] Debener S., Ullsperger M., Siegel M., Fiehler K., von Cramon D.Y., Engel A.K. (2005). Trial-by-trial coupling of concurrent electroencephalogram and functional magnetic resonance imaging identifies the dynamics of performance monitoring. J. Neurosci..

[bb0075] Debener S., Ullsperger M., Siegel M., Engel A.K. (2006). Single-trial EEG-fMRI reveals the dynamics of cognitive function. Trends Cogn. Sci..

[bb0085] Delorme A., Makeig S. (2004). EEGLAB: an open source toolbox for analysis of single-trial EEG dynamics including independent component analysis. J. Neurosci. Methods.

[bb0090] Dien J. (1998). Addressing misallocation of variance in principal components analysis of event-related potentials. Brain Topogr..

[bb0095] Dien J. (2010). Evaluating two-step PCA of ERP data with Geomin, Infomax, Oblimin, Promax, and Varimax rotations. Psychophysiology.

[bb0100] Dien J., Khoe W., Mangun G.R. (2007). Evaluation of PCA and ICA of simulated ERPs: Promax vs. Infomax rotations. Hum. Brain Mapp..

[bb0105] Fabiani M., Gratton G., Karis D., Donchin E., Ackles P.K., Jennings J.R., Coles M.G.H. (1987). Definition, identification, and reliability of measurement of the P300 component of the event-related brain potential. Advances in Psychophysiology.

[bb0110] Friman O., Borga M., Lundberg P., Knutsson H. (2003). Adaptive analysis of fMRI data. Neuroimage.

[bb0115] Frot M., Mauguiere F., Magnin M., Garcia-Larrea L. (2008). Parallel processing of nociceptive A-delta inputs in SII and midcingulate cortex in humans. J. Neurosci..

[bb0120] Garcia-Larrea L., Frot M., Valeriani M. (2003). Brain generators of laser-evoked potentials: from dipoles to functional significance. Neurophysiol. Clin..

[bb0125] Hossein-Zadeh G.A., Ardekani B.A., Soltanian-Zadeh H. (2003). A signal subspace approach for modeling the hemodynamic response function in fMRI. Magn. Reson. Imaging.

[bb0140] Hu L., Mouraux A., Hu Y., Iannetti G.D. (2010). A novel approach for enhancing the signal-to-noise ratio and detecting automatically event-related potentials (ERPs) in single trials. Neuroimage.

[bb0130] Hu L., Liang M., Mouraux A., Wise R.G., Hu Y., Iannetti G.D. (2011). Taking into account latency, amplitude and morphology: improved estimation of single-trial ERPs by wavelet filtering and multiple linear regression. J. Neurophysiol..

[bb0155] Hu L., Zhang Z.G., Hung Y.S., Luk K.D., Iannetti G.D., Hu Y. (2011). Single-trial detection of somatosensory evoked potentials by probabilistic independent component analysis and wavelet filtering. Clin. Neurophysiol..

[bb0145] Hu L., Peng W., Valentini E., Zhang Z., Hu Y. (2013). Functional features of nociceptive-induced suppression of alpha band electroencephalographic oscillations. J. Pain.

[bb0150] Hu L., Xiao P., Zhang Z.G., Mouraux A., Iannetti G.D. (2014). Single-trial time-frequency analysis of electrocortical signals: baseline correction and beyond. Neuroimage.

[bb0160] Huang G., Xiao P., Hung Y.S., Zhang Z.G., Hu L. (2013). A novel approach to predict subjective pain perception from single-trial laser-evoked potentials. Neuroimage.

[bb0165] Hyvarinen A. (1999). Fast and robust fixed-point algorithms for independent component analysis. IEEE Trans. Neural Netw..

[bb0170] Hyvarinen A., Oja E. (2000). Independent component analysis: algorithms and applications. Neural Netw..

[bb0180] Iannetti G.D., Mouraux A., Mulert C., Lemieux L. (2009). Combining EEG and fMRI in pain research. EEG-fMRI: Physiological Basis, Technique, and Applications.

[bb0185] Iannetti G.D., Truini A., Galeotti F., Romaniello A., Manfredi M., Cruccu G. (2001). Usefulness of dorsal laser evoked potentials in patients with spinal cord damage: report of two cases. J. Neurol. Neurosurg. Psychiatry.

[bb0190] Iannetti G.D., Truini A., Romaniello A., Galeotti F., Rizzo C., Manfredi M., Cruccu G. (2003). Evidence of a specific spinal pathway for the sense of warmth in humans. J. Neurophysiol..

[bb0195] Iannetti G.D., Zambreanu L., Cruccu G., Tracey I. (2005). Operculoinsular cortex encodes pain intensity at the earliest stages of cortical processing as indicated by amplitude of laser-evoked potentials in humans. Neuroscience.

[bb0205] Iannetti G.D., Zambreanu L., Wise R.G., Buchanan T.J., Huggins J.P., Smart T.S., Vennart W., Tracey I. (2005). Pharmacological modulation of pain-related brain activity during normal and central sensitization states in humans. Proc. Natl. Acad. Sci. U. S. A..

[bb0200] Iannetti G.D., Zambreanu L., Tracey I. (2006). Similar nociceptive afferents mediate psychophysical and electrophysiological responses to heat stimulation of glabrous and hairy skin in humans. J. Physiol..

[bb0175] Iannetti G.D., Hughes N.P., Lee M.C., Mouraux A. (2008). Determinants of laser-evoked EEG responses: pain perception or stimulus saliency?. J. Neurophysiol..

[bb0210] Jensen M.P., Karoly P., Turk D.C., Melzack R. (2001). Self-report scales and procedures for assessing pain in adults. Handbook of Pain Assessment.

[bb0215] Jolliffe I.T. (2002). Principal Component Analysis.

[bb0220] Jongsma M.L.A., Eichele T., Van Rijn C.M., Coenen A.M.L., Hugdahl K., Nordby H., Quiroga R.Q. (2006). Tracking pattern learning with single-trial event-related potentials. Clin. Neurophysiol..

[bb0225] Jung T.P., Makeig S., Westerfield M., Townsend J., Courchesne E., Sejnowski T.J. (2001). Analysis and visualization of single-trial event-related potentials. Hum. Brain Mapp..

[bb0230] Kaiser H.F. (1958). The varimax criterion for analytic rotation in factor analysis. Psychometrika..

[bb0235] Kakigi R., Shibasaki H., Ikeda A. (1989). Pain-related somatosensory evoked potentials following CO2 laser stimulation in man. Electroencephalogr. Clin. Neurophysiol..

[bb0240] Kayser J., Tenke C.E. (2003). Optimizing PCA methodology for ERP component identification and measurement: theoretical rationale and empirical evaluation. Clin. Neurophysiol..

[bb0245] Lachaux J.P., Rodriguez E., Martinerie J., Varela F.J. (1999). Measuring phase synchrony in brain signals. Hum. Brain Mapp..

[bb0250] Lee M.C., Mouraux A., Iannetti G.D. (2009). Characterizing the cortical activity through which pain emerges from nociception. J. Neurosci..

[bb0255] Legrain V., Bruyer R., Guerit J.M., Plaghki L. (2003). Nociceptive processing in the human brain of infrequent task-relevant and task-irrelevant noxious stimuli. A study with event-related potentials evoked by CO2 laser radiant heat stimuli. Pain.

[bb0260] Liu Y., Huang H., McGinnis-Deweese M., Keil A., Ding M. (2012). Neural substrate of the late positive potential in emotional processing. J. Neurosci..

[bb0265] Makeig S., Jung T.P., Bell A.J., Ghahremani D., Sejnowski T.J. (1997). Blind separation of auditory event-related brain responses into independent components. Proc. Natl. Acad. Sci. U. S. A..

[bb0270] Makinen V.T., May P.J., Tiitinen H. (2004). Human auditory event-related processes in the time-frequency plane. Neuroreport.

[bb0280] Mayhew S.D., Iannetti G.D., Woolrich M.W., Wise R.G. (2006). Automated single-trial measurement of amplitude and latency of laser-evoked potentials (LEPs) using multiple linear regression. Clin. Neurophysiol..

[bb0275] Mayhew S.D., Dirckx S.G., Niazy R.K., Iannetti G.D., Wise R.G. (2010). EEG signatures of auditory activity correlate with simultaneously recorded fMRI responses in humans. Neuroimage.

[bb0285] Mitsis G.D., Iannetti G.D., Smart T.S., Tracey I., Wise R.G. (2008). Regions of interest analysis in pharmacological fMRI: how do the definition criteria influence the inferred result?. Neuroimage.

[bb0290] Motulsky H., Christopoulos A. (2004). Fitting Models to Biological Data Using Linear and Nonlinear Regression: A Practical Guide to Curve Fitting.

[bb0295] Mountcastle V.B. (1998). Perceptual Neuroscience: The Cerebral Cortex.

[bb0305] Mouraux A., Iannetti G.D. (2008). Across-trial averaging of event-related EEG responses and beyond. Magn. Reson. Imaging.

[bb0310] Mouraux A., Iannetti G.D. (2009). Nociceptive laser-evoked brain potentials do not reflect nociceptive-specific neural activity. J. Neurophysiol..

[bb0315] Mouraux A., Plaghki L. (2004). Single-trial detection of human brain responses evoked by laser activation of Adelta-nociceptors using the wavelet transform of EEG epochs. Neurosci. Lett..

[bb0300] Mouraux A., Guerit J.M., Plaghki L. (2003). Non-phase locked electroencephalogram (EEG) responses to CO2 laser skin stimulations may reflect central interactions between A partial partial differential- and C-fibre afferent volleys. Clin. Neurophysiol..

[bb0320] Mu Y., Fan Y., Mao L., Han S. (2008). Event-related theta and alpha oscillations mediate empathy for pain. Brain Res..

[bb0325] Neuper C., Klimesch W. (2006). Event-related Dynamics of Brain Oscillations.

[bb0330] Nunez P.L., Srinivasan R. (2006). Electric Fields of the Brain: The Neurophysics of EEG.

[bb0335] Ohara S., Crone N.E., Weiss N., Lenz F.A. (2004). Attention to a painful cutaneous laser stimulus modulates electrocorticographic event-related desynchronization in humans. Clin. Neurophysiol..

[bb0340] Orssaud C. (2003). Leber's hereditary optic neuropathy. Orphanet Encyclopedia.

[bb0345] Pelosi L., Geesken J.M., Holly M., Hayward M., Blumhardt L.D. (1997). Working memory impairment in early multiple sclerosis. Evidence from an event-related potential study of patients with clinically isolated myelopathy. Brain.

[bb0350] Pfurtscheller G., Lopes da Silva F.H. (1999). Event-related EEG/MEG synchronization and desynchronization: basic principles. Clin. Neurophysiol..

[bb0355] Pfurtscheller G., Neuper C. (1994). Event-related synchronization of mu rhythm in the EEG over the cortical hand area in man. Neurosci. Lett..

[bb0360] Ploner M., Gross J., Timmermann L., Pollok B., Schnitzler A. (2006). Pain suppresses spontaneous brain rhythms. Cereb. Cortex.

[bb0365] Porcaro C., Barbati G., Zappasodi F., Rossini P.M., Tecchio F. (2008). Hand sensory-motor cortical network assessed by functional source separation. Hum. Brain Mapp..

[bb0370] Porcaro C., Coppola G., Di Lorenzo G., Zappasodi F., Siracusano A., Pierelli F., Rossini P.M., Tecchio F., Seri S. (2009). Hand somatosensory subcortical and cortical sources assessed by functional source separation: an EEG study. Hum. Brain Mapp..

[bb0375] Porcaro C., Ostwald D., Bagshaw A.P. (2010). Functional source separation improves the quality of single trial visual evoked potentials recorded during concurrent EEG-fMRI. Neuroimage.

[bb0380] Purves A.M., Boyd S.G. (1993). Time-shifted averaging for laser evoked potentials. Electroencephalogr. Clin. Neurophysiol..

[bb0385] Quiroga R.Q. (2000). Obtaining single stimulus evoked potentials with wavelet denoising. Physica D.

[bb0390] Quiroga R.Q., Garcia H. (2003). Single-trial event-related potentials with wavelet denoising. Clin. Neurophysiol..

[bb0395] Richman M.B. (1986). Rotation of principal components. J. Climatol..

[bb0400] Rodriguez E., George N., Lachaux J.P., Martinerie J., Renault B., Varela F.J. (1999). Perception's shadow: long-distance synchronization of human brain activity. Nature.

[bb0405] Schulz E., Tiemann L., Schuster T., Gross J., Ploner M. (2011). Neurophysiological coding of traits and states in the perception of pain. Cereb. Cortex.

[bb0410] Spencer K.M., Handy T.C. (2005). Averaging, detection, and classification of single-trial ERPs. Event-related Potentials: A Methods Handbook.

[bb0415] Stancak A., Svoboda J., Rachmanova R., Vrana J., Kralik J., Tintera J. (2003). Desynchronization of cortical rhythms following cutaneous stimulation: effects of stimulus repetition and intensity, and of the size of corpus callosum. Clin. Neurophysiol..

[bb0420] Tallon-Baudry C., Bertrand O., Wienbruch C., Ross B., Pantev C. (1997). Combined EEG and MEG recordings of visual 40 Hz responses to illusory triangles in human. Neuroreport.

[bb0425] Tang A.C., Sutherland M.T., McKinney C.J. (2005). Validation of SOBI components from high-density EEG. Neuroimage.

[bb0430] Tecchio F., Porcaro C., Barbati G., Zappasodi F. (2007). Functional source separation and hand cortical representation for a brain-computer interface feature extraction. J. Physiol..

[bb0435] Treede R.D., Lorenz J., Baumgartner U. (2003). Clinical usefulness of laser-evoked potentials. Neurophysiol. Clin..

[bb0440] Valentini E., Torta D.M., Mouraux A., Iannetti G.D. (2011). Dishabituation of laser-evoked EEG responses: dissecting the effect of certain and uncertain changes in stimulus modality. J. Cogn. Neurosci..

[bb0445] Zhang Z.G., Hu L., Hung Y.S., Mouraux A., Iannetti G.D. (2012). Gamma-band oscillations in the primary somatosensory cortex—a direct and obligatory correlate of subjective pain intensity. J. Neurosci..

