# Supplementary material for: Multiple linear regression to estimate time-frequency electrophysiological responses in single trials
Source: Neuroimage. 2015 May 1;111:442–53. doi: 10.1016/j.neuroimage.2015.01.062 (PMC4401443; doi:10.1016/j.neuroimage.2015.01.062)
Supplement: Supplementary file 1 — Supplementary Materials. [file mmc1.docx]

1. Effect of PC thresholding on single-trial performance

The TFD of each principal component (PC), isolated using principal component analysis with Varimax rotation, was thresholded using a cut-off at two standard deviations from the mean of all time-frequency points ([Mayhew et al., 2010](#_ENREF_5)). Therefore, only the time-frequency points with amplitudes above (ERP and ERS) or below (ERD) two standard deviations from the mean were preserved. The value of all other time-frequency points was set to zero ([Mayhew et al., 2010](#_ENREF_5)). The objective of this operation was to null the low amplitude signal changes, which were treated as noise in the TFDs.

We assessed the effect of TFD thresholding using different cut-off levels on the performance of single-trial estimation, as follows. We first compared the thresholded features using different levels of cut-off (from 0 to 3 SDs). As displayed in Supplementary Fig. 1, the size of the surviving time-frequency regions that captured the signal-of-interest was markedly decreased with increasing cut-off levels. Using the 1-SD cut-off, there were several additional clusters in the TFDs of ERD and ERS (marked in purple). It should be noted that these clusters (e.g., the negative blob in the TFD of ERS located at 900-1000 ms and 8-15 Hz) showed remarkable differences with the dominant feature in terms of polarity and time-frequency distribution. Second, we performed the correlation analysis between the estimated single-trial magnitudes obtained using different levels of thresholding in TF-MLR (similar performance was obtained using TF-MLR_d_). All these correlations were extremely significant (Supplementary Table 1; p<0.001 for all correlations). In other words, the obtained single-trial parameters were markedly similar even the level of thresholding was different, indicating that the cut-off level does not seem to be a key parameter affecting the performance of the single-trial estimation.


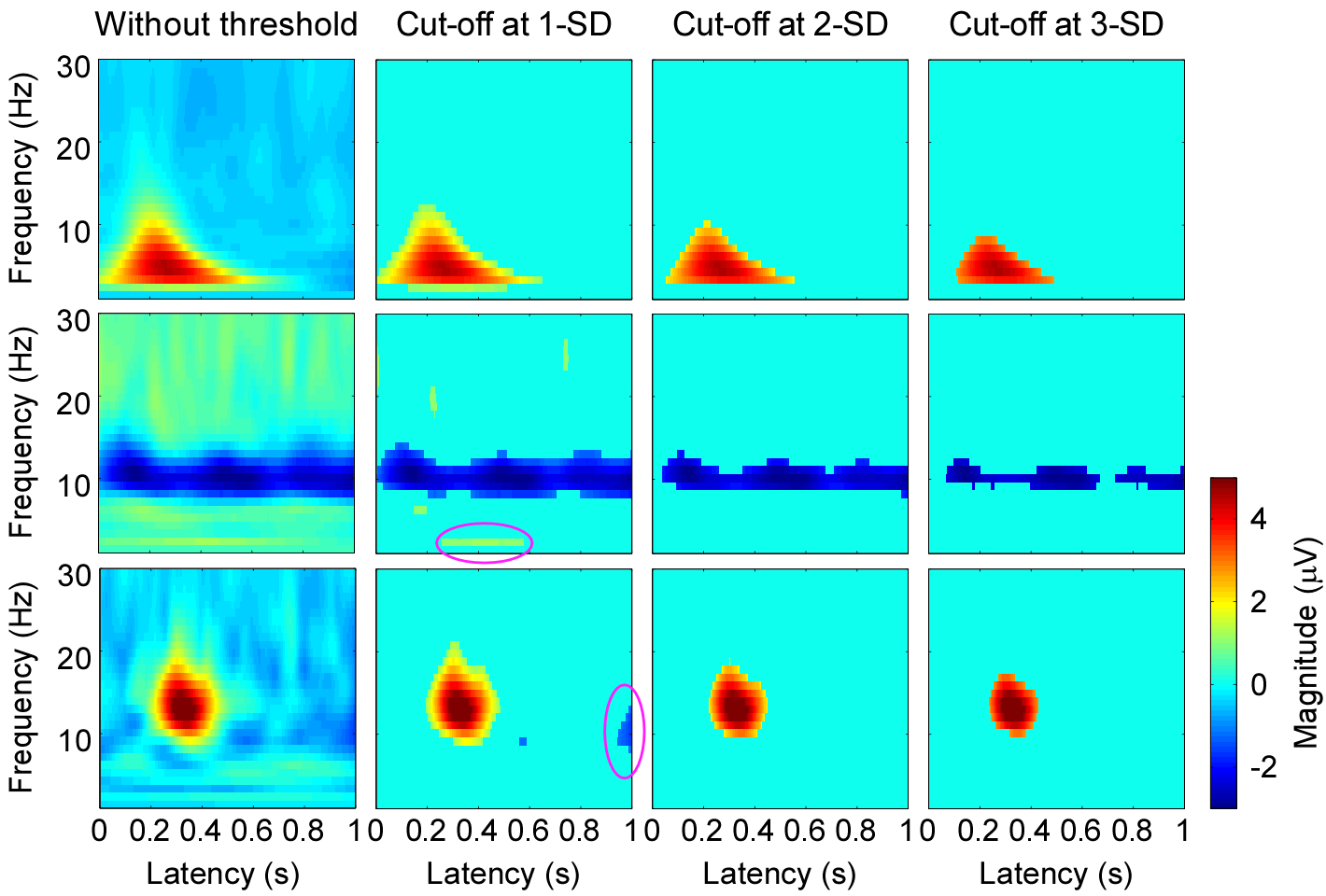


**Supplementary Fig. 1.** Effect of different thresholding cut-off on the isolation of the time-frequency features. With the increase of thresholding (from 0-SD to 3-SD), the size of the signal-of-interest was decreased. Note that when a cut-off at 1-SD was used, there were some noise blobs (marked in purple) in the TFDs of ERD and ERS.

**Supplementary Table 1.** Correlation coefficients between single-trial magnitudes obtained using different levels of thresholding in TF-MLR (0-SD: without thresholding; 1-SD, 2-SD, and 3-SD: thresholding using a cut-off of one, two, and three standard deviations, respectively). All correlations are highly significant (p<0.001).

|  | 0-SD & 1-SD | 0-SD & 2-SD | 0-SD & 3-SD | 1-SD & 2-SD | 1-SD & 3-SD | 2-SD & 3-SD |
| --- | --- | --- | --- | --- | --- | --- |
| ERP magnitude | R=0.904 | R=0.879 | R=0.865 | R=0.988 | R=0.976 | R=0.992 |
| ERD magnitude | R=0.964 | R=0.955 | R=0.939 | R=0.981 | R=0.955 | R=0.977 |
| ERS magnitude | R=0.903 | R=0.882 | R=0.875 | R=0.973 | R=0.952 | R=0.984 |

2. Assessment of the performance of TF-MLR and TF-MLR_d_ using a simulated dataset

Based on the time-frequency features isolated by PCA with Varimax rotation (right panel of Fig. 1 in the main text), a new, simulated dataset was generated by: (1) modeling the trial-by-trial variability of amplitude, latency, frequency, morphology in the time and frequency domains for each time-frequency features; and (2) adding different levels of background EEG noise (Supplementary Fig. 2).

**2.1 Modeling the trial-by-trial variability**

Similarly to previous studies ([Hu et al., 2011](#_ENREF_2); [Spencer, 2005](#_ENREF_6)), the variability of single-trial amplitude was modeled by multiplying the isolated time-frequency features by a range of positive coefficients (randomly distributed from 0.5 to 1.5). The variability of single-trial latency and frequency was respectively modeled by shifting the isolated time-frequency features along the time and frequency axis with random latency (within ±100 ms) and frequency (within ±2 Hz) values. The variability of single-trial morphology in time and frequency domains was respectively modeled by compressing the template along the time and frequency axis (randomly distributed from 1 to 1.5 times for both domains) centered at the peak of each time-frequency feature. The time-frequency features (ERP/ERD/ERS) with added variability were summed to generate a complete, simulated single-trial TFD (Supplementary Fig. 2). This procedure was repeated 577 times, to generate a modeled dataset with the same number of trials of the resting EEG dataset. Note that the simulated dataset with variability in amplitude, latency, and frequency was labeled as ‘dataset #1’, and the simulated dataset with all variability factors (i.e., amplitude, latency, frequency, latency compression, frequency compression) was labeled as ‘dataset #2’.

**2.2 Adding background noise**

TFDs of real resting EEG data, obtained from the same population (577 epochs in total), were used to provide background noise. To assess the performance of time-frequency single-trial methods at different noise levels, the resting EEG data were multiplied by 4 different weights to ensure that the signal-to-noise ratio of the simulated datasets was 0.2, 0.5, 1, and 2 respectively. These background noise trials of each level were added to the simulated datasets (one noise trial for each simulated trial), thus generating four simulated datasets, each with a different level of background noise. The SNR of the simulated datasets was estimated as follows ([Iyer and Zouridakis, 2007](#_ENREF_4); [Zouridakis et al., 1997](#_ENREF_7)):

$SNR=\frac{\sigma_{TFD_{i}}^{2}}{\sigma_{EEG_{i}}^{2}}$ (1)

where $i$ is the trial number,$\sigma_{\mathrm{ERP}_{i}}^{2}$ is the variance of the simulated *i*th TFD resulting from the addition of variations to the isolated time-frequency features (e.g., Supplementary Fig. 2, second and third columns of the fourth row), and$\sigma_{\mathrm{EEG}_{i}}^{2}$ is the variance of the *i*th EEG noise (e.g., Supplementary Fig. 2, first column of the fourth row).

The performance of TF-MLR and TF-MLR_d_ was assessed by calculating the absolute difference between the original and the estimated single-trial parameters (i.e., the latency, frequency, and magnitude of ERP/ERD/ERS of each single trial). For each level of noise (SNR=0.2, SNR=0.5, SNR=1, and SNR=2) and each dataset (#1 and #2), the absolute differences obtained using TF-MLR and TF-MLR_d_ were compared using a paired-sample t test. To account for multiple comparisons, the obtained significances were adjusted using Bonferroni correction.

Supplementary Fig. 3 shows the performance of TF-MLR and TF-MLR_d_. Regardless of tested datasets (#1 or #2), TF-MLR_d_ showed a better performance than TF-MLR in estimating ERP latency and ERS frequency for all levels of SNR (p < 0.05 after Bonferroni correction, the same hereinafter). In contrast, TF-MLR demonstrated a better performance than TF-MLR_d_ in estimating ERD latency, but only at low levels of SNR. Overall, TF-MLR_d_ improved the performance of single-trial magnitude estimation when the level of SNR was high, while TF-MLR provided a more accurate single-trial magnitude estimation when the level of SNR was low.


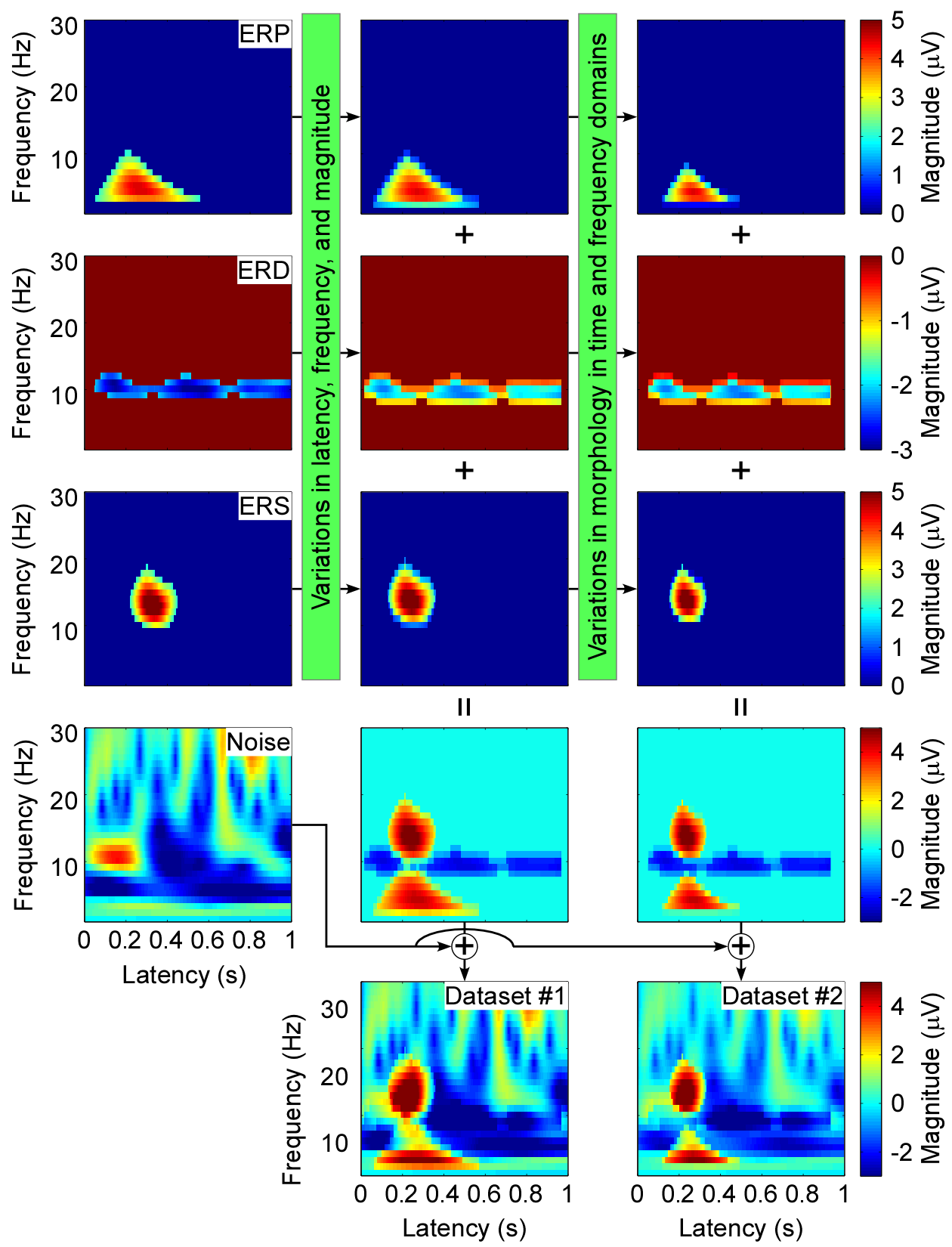


**Supplementary Fig. 2.** Flowchart describing the generation of the simulation datasets with different trial-by-trial variability. Simulated data with variability in amplitude, latency, and frequency were labeled as dataset #1, and simulated data with all variability factors (i.e., amplitude, latency, frequency, latency compression, frequency compression) were labeled as dataset #2.


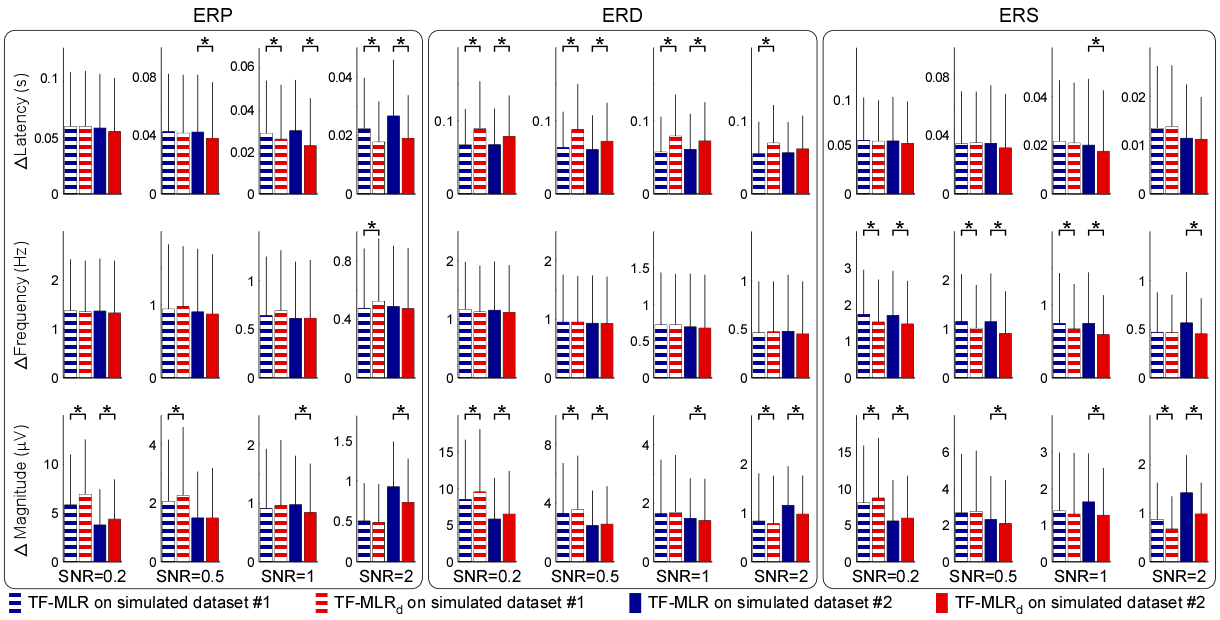


**Supplementary Fig. 3.** Performance of TF-MLR and TF-MLR_d_ approaches to estimate single-trial ERP/ERD/ERS parameters at different levels of SNR. Vertical error bars represent standard deviation across all trials; asterisks (*) indicates significant differences (P < 0.05, paired-sample t-test with Bonferroni correction).

Altogether, the simulation study indicated that the TF-MLR approach, which uses fewer regressors than the TF-MLR_d_ approach, is more *specific* in capturing stimulus-related responses ([Friman et al., 2003](#_ENREF_1)), but is unable to capture the variability of the response morphology, both in time and frequency domains. Therefore, the TF-MLR approach provides a simple and robust single-trial estimate, and it is particularly appropriate when dealing with EEG responses with relatively low SNR (for example the early N1 wave of LEPs; Hu et al., 2010)(Hu et al., 2010). In contrast, the higher number of regressors in the TF-MLR_d_ approach allows a higher *sensitivity* in detecting the response variability ([Hu et al., 2011](#_ENREF_2)), but with the possible drawback of fitting some noise. Therefore, the TF-MLR_d_ approach provides a more accurate estimate of single-trial responses with relatively high SNR (e.g., intracranial recordings, interictal waves recorded from epilepsy patients).

References

Friman, O., Borga, M., Lundberg, P., Knutsson, H., 2003. Adaptive analysis of fMRI data. Neuroimage 19, 837-845.

Hu, L., Liang, M., Mouraux, A., Wise, R.G., Hu, Y., Iannetti, G.D., 2011. Taking into account latency, amplitude, and morphology: improved estimation of single-trial ERPs by wavelet filtering and multiple linear regression. J Neurophysiol 106, 3216-3229.

Hu, L., Mouraux, A., Hu, Y., Iannetti, G.D., 2010. A novel approach for enhancing the signal-to-noise ratio and detecting automatically event-related potentials (ERPs) in single trials. Neuroimage 50, 99-111.

Iyer, D., Zouridakis, G., 2007. Single-trial evoked potential estimation: comparison between independent component analysis and wavelet denoising. Clin Neurophysiol 118, 495-504.

Mayhew, S.D., Dirckx, S.G., Niazy, R.K., Iannetti, G.D., Wise, R.G., 2010. EEG signatures of auditory activity correlate with simultaneously recorded fMRI responses in humans. Neuroimage 49, 849-864.

Spencer, K.M., 2005. Averaging, Detection, and Classification of Single-Trial ERPs. In: Handy, T.C. (Ed.), Event-related potentials : a methods handbook. MIT Press, Cambridge, Mass., pp. 209-227.

Zouridakis, G., Jansen, B.H., Boutros, N.N., 1997. A fuzzy clustering approach to EP estimation. IEEE Trans Biomed Eng 44, 673-680.
